# Thoracic and cardiovascular surgeries in Japan during 2018: Annual report by the Japanese Association for Thoracic Surgery

**DOI:** 10.1007/s11748-020-01460-w

**Published:** 2020-10-22

**Authors:** Committee for Scientific Affairs, The Japanese Association for Thoracic Surgery, Hideyuki Shimizu, Morihito Okada, Yasushi Toh, Yuichiro Doki, Shunsuke Endo, Hirotsugu Fukuda, Yasutaka Hirata, Hisashi Iwata, Junjiro Kobayashi, Hiraku Kumamaru, Hiroaki Miyata, Noboru Motomura, Shoji Natsugoe, Soji Ozawa, Yoshikatsu Saiki, Aya Saito, Hisashi Saji, Yukio Sato, Tsuyoshi Taketani, Kazuo Tanemoto, Akira Tangoku, Wataru Tatsuishi, Hiroyuki Tsukihara, Masayuki Watanabe, Hiroyuki Yamamoto, Kenji Minatoya, Kohei Yokoi, Yutaka Okita, Masanori Tsuchida, Yoshiki Sawa

**Affiliations:** 1 Committee for Scientific Affairs, The Japanese Association for Thoracic Surgery, Tokyo, Japan; 2 Department of Cardiovascular Surgery, Keio University, 35, Shinanomachi, Shinjuku-ku, Tokyo, Japan; 3 Department of Surgical Oncology, Hiroshima University, Hiroshima, Japan; 4 Department of Gastroenterological Surgery, National Hospital Organization Kyushu Cancer Center, Fukuoka, Japan; 5 Department of Gastroenterological Surgery, Osaka University, Suita, Osaka Japan; 6 Department of Thoracic Surgery, Jichi Medical University, Shimotsuke, Japan; 7 Department of Cardiac and Vascular Surgery, Dokkyo Medical University School of Medicine, Shimotsuga-gun, Tochigi, Japan; 8 Department of Cardiac Surgery, The University of Tokyo Hospital, Tokyo, Japan; 9 Department of General Thoracic Surgery, Gifu University Hospital, Gifu, Japan; 10 Department of Cardiovascular Surgery, National Cerebral and Cardiovascular Center, Suita, Japan; 11 Department of Healthcare Quality Assessment, Graduate School of Medicine, University of Tokyo, Tokyo, Japan; 12 Department of Health Policy and Management, Keio University, Tokyo, Japan; 13 Department of Cardiovascular Surgery, Toho University Sakura Medical Center, Sakura, Japan; 14 Department of Digestive Surgery, Breast and Thyroid Surgery, Kagoshima University Graduate School of Medicine, Kagoshima, Japan; 15 Department of Gastroenterological Surgery, Tokai University School of Medicine, Isehara, Japan; 16 Division of Cardiovascular Surgery, Tohoku University Graduate School of Medicine, Sendai, Japan; 17 Department of Cardiovascular Surgery, Toho University Sakura Medical Center, Sakura, Japan; 18 Department of Chest Surgery, St. Marianna University School of Medicine, Kawasaki, Japan; 19 Department of Thoracic Surgery, University of Tsukuba, Tsukuba, Japan; 20 Department of Cardiovascular Surgery, Mitsui Memorial Hospital, Tokyo, Japan; 21 Department of Cardiovascular Surgery, Kawasaki Medical School, Kurashiki, Japan; 22 Department of Thoracic, Endocrine Surgery and Oncology, Institute of BioMedicine, Tokushima University Graduate School, Tokushima, Japan; 23 Division of Cardiovascular Surgery, Department of General Surgical Science, Gunma University, Maebashi, Japan; 24 Department of Cardiac Surgery, The University of Tokyo Hospital, Tokyo, Japan; 25 Department of Gastroenterological Surgery, Cancer Institute Hospital of Japanese Foundation for Cancer Research, Tokyo, Japan; 26 Department of Health Policy and Management, Keio University, Tokyo, Japan; 27 Department of Cardiovascular Surgery, Graduate School of Medicine, Kyoto University, Kyoto, Japan; 28 Chunichi Hospital, Nagoya, Japan; 29 Cardio-Aortic Center, Takatsuki General Hospital, Takatsuki, Japan; 30 Division of Thoracic and Cardiovascular Surgery, Niigata University Graduate School of Medical and Dental Sciences, Niigata, Japan; 31 Department of Cardiovascular Surgery, Graduate School of Medicine, Osaka University, Osaka, Japan

The Japanese Association for Thoracic Surgery has conducted annual surveys of thoracic surgery throughout Japan since 1986 to determine statistics pertaining to the number of procedures performed according to surgical categories. We herein summarize the results of the association’s annual survey of thoracic surgeries performed in 2018.

Adhering to the norm thus far, thoracic surgery had been classified into three categories, cardiovascular, general thoracic, and esophageal surgeries, with patient data for each group being examined and analyzed. We honor and value all members’ continued professional support and contributions.

Incidence of hospital mortality was included in the survey to determine nationwide status, which has contributed to Japanese surgeons’ understanding of the present status of thoracic surgery in Japan while helping to effect improvements in surgical outcomes by enabling comparisons between their work and that of others. This approach has enabled the association to gain a better understanding of present problems and future prospects, which is reflected in its activities and member education.

Thirty-day mortality (otherwise known as *operative mortality*) is defined as death within 30 days of surgery, regardless of the patient's geographic location, including post-discharge from the hospital. *Hospital mortality* is defined as death within any time interval following surgery among patients yet to be discharged from the hospital.

While hospital-to-hospital transfer during esophageal surgery is not considered a form of discharge, transfer to a nursing home or a rehabilitation unit *is* considered hospital discharge, unless the patient subsequently dies of complications from surgery. In contrast, hospital-to-hospital transfer 30 days following cardiovascular and general thoracic surgeries is considered discharge given that data related to the National Clinical Database (NCD) were employed in these categories.

## Survey abstract

All data pertaining to cardiovascular and thoracic surgeries were obtained from the NCD, whereas data regarding esophageal surgery were collected from a survey questionnaire derived from the Japanese Association for Thoracic Surgery documentation. This is because NCD information regarding esophageal surgery does not include non-surgical cases (i.e., patients with adjuvant chemotherapy or radiation only).

Given the changes in data collection related to cardiovascular surgery [initially self-reported using questionnaire sheets in each participating institution up to 2014, followed by downloading of an automatic package from the Japanese Cardiovascular Surgery Database (JCVSD), a cardiovascular subsection of the NCD], response rates were unavailable and were therefore not indicated in the cardiovascular surgery category (Table 1). Additionally, the number of institutions (based on surgery count) was not calculated in the cardiovascular surgery category (Table 2).

**Table 1:** Number of institutions involved in the survey

	Questionnaires		
	Sent out	Responded	Response rate
(A) Cardiovascular surgery			
(B) General Thoracic Surgery	749	676	90.3%
(C) Esophageal surgery		552

**Table 2:** Categories subclassified according to the number of operations performed

Number of operations performed	Category
	General thoracic surgery
0	5
1–24	38
25–49	94
50–99	193
100–149	121
150–199	107
≥ 200	118
Total	676

## Final report: 2018

### (A) Cardiovascular surgery

We are extremely pleased with the cooperation of our colleagues (members) in completing the cardiovascular surgery survey, which has undoubtedly improved the quality of this annual report. We are truly grateful for the significant efforts made by all participants within each participating institution in completing the JCVSD/NCD.

**Fig. 1:**
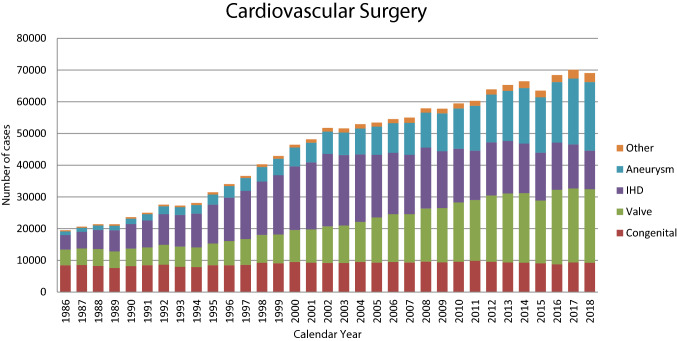
Cardiovascular surgery.* IHD* ischemic heart disease

Figure 1 illustrates the development of cardiovascular surgery in Japan over the past 32 years. Aneurysm surgery includes only surgeries for thoracic and thoracoabdominal aortic aneurysms. Extra-anatomic bypass surgery for thoracic aneurysm and pacemaker implantation have been excluded from the survey since 2015. Assist device implantations were not included in the total number of surgical procedures but were nonetheless included in the survey.

A total of 69,063 cardiovascular surgeries, including 51 heart transplants, had been performed in 2018, a decrease of 0.7% compared to that in 2017 (n = 70,078).

Compared to data for 2017 [1] and 2008 [2], data for 2018 showed 1.2% (9253 vs. 9368) and 3.6% fewer surgeries for congenital heart disease, 0.5% (23,205 vs. 23,312) fewer and 38.6% more surgeries for valvular heart disease, 12.7% (12,135 vs. 13,898) and 36.9% fewer surgeries for ischemic heart procedures, and 4.2% (21,624 vs. 20,746) and 96.6% more surgeries for thoracic aortic aneurysm, respectively. Data for individual categories are summarized in Tables 3, 4, 5, 6, 7, 8.

**Table 3:** Congenital (total; 9368) (1) CPB ( +) (total; 7130)

	Neonate				Infant				1–17 years				≥ 18 years				Total			
	Cases	30-Day mortality		Hospital mortality	Cases	30-Day mortality		Hospital mortality	Cases	30-Day mortality		Hospital mortality	Cases	30-Day mortality		Hospital mortality	Cases	30-day mortality		Hospital mortality
		Hospital	After discharge			Hospital	After discharge			Hospital	After discharge			Hospital	After discharge			Hospital	After discharge	
PDA	1	0	0	0	8	0	0	1 (12.5)	6	0	0	0	19	1 (5.3)	0	1 (5.3)	34	1 (2.9)	0	2 (5.9)
Coarctation (simple)	4	0	0	0	14	0	0	0	10	0	0	0	7	0	0	0	35	0	0	0
+ VSD	43	1 (2.3)	0	1 (2.3)	41	1 (2.4)	0	1 (2.4)	12	0	0	0	1	0	0	0	97	2 (2.1)	0	2(2.1)
+ DORV	3	0	0	0	0	0	0	0	0	0	0	0	0	0	0	0	3	0	0	0
+ AVSD	4	0	0	0	1	0	0	0	0	0	0	0	0	0	0	0	5	0	0	0
+ TGA	0	0	0	0	1	0	0	0	0	0	0	0	0	0	0	0	1	0	0	0
+ SV	2	0	0	0	0	0	0	0	0	0	0	0	0	0	0	0	2	0	0	0
+ Others	5	0	0	0	6	0	0	0	12	0	0	1 (8.3)	0	0	0	0	23	0	0	1 (4.3)
Interrupt. of Ao (simple)	0	0	0	0	0	0	0	0	0	0	0	0	0	0	0	0	0	0	0	0
+ VSD	21	1 (4.8)	0	1 (4.8)	35	1 (2.9)	0	1 (2.9)	19	0	0	0	0	0	0	0	75	2 (2.7)	0	2 (2.7)
+ DORV	0	0	0	0	0	0	0	0	0	0	0	0	0	0	0	0	0	0	0	0
+ Truncus	3	0	0	0	6	0	0	0	2	0	0	0	0	0	0	0	11	0	0	0
+ TGA	1	0	0	0	0	0	0	0	0	0	0	0	0	0	0	0	1	0	0	0
+ Others	2	0	0	0	1	0	0	0	1	0	0	0	0	0	0	0	4	0	0	0
Vascular ring	1	0	0	0	3	0	0	0	0	0	0	0	0	0	0	0	4	0	0	0
PS	2	0	0	0	26	0	0	0	57	0	0	0	24	0	0	0	109	0	0	0
PA∙IVS or Critical PS	14	0	0	0	60	1 (1.7)	0	1 (1.7)	59	0	0	0	7	0	0	0	140	1 (0.7)	0	1 (0.7)
TAPVR	117	6 (5.1)	0	14 (12.0)	75	3 (4.0)	0	4 (5.3)	18	0	0	1 (5.6)	1	0	0	0	211	9 (4.3)	0	19 (9.0)
PAPVR ± ASD	0	0	0	0	6	0	0	0	39	0	0	0	11	0	0	0	56	0	0	0
ASD	1	0	0	0	51	0	0	0	528	0	0	0	822	13 (1.6)	0	13 (1.6)	1402	13 (0.9)	0	13 (0.9)
Cor triatriatum	0	0	0	0	12	0	0	1 (8.3)	5	0	0	0	0	0	0	0	17	0	0	1 (5.9)
AVSD (partial)	1	0	0	0	7	0	0	0	37	0	0	0	9	0	0	0	54	0	0	0
AVSD (complete)	6	0	0	1 (16.7)	106	0	0	1 (0.9)	86	1 (1.2)	0	3 (3.5)	5	0	0	0	203	1 (0.5)	0	5 (2.5)
+ TOF or DORV	0	0	0	0	8	0	0	1 (12.5)	7	0	0	0	2	0	0	0	17	0	0	1 (5.9)
+ Others	0	0	0	0	0	0	0	0	0	0	0	0	0	0	0	0	0	0	0	0
VSD (subarterial)	3	0	0	0	94	1 (1.1)	0	1 (1.1)	169	0	0	0	5	0	0	0	271	1 (0.4)	0	1 (0.4)
VSD (perimemb./muscular)	15	0	0	0	706	0	0	0	365	0	0	1 (0.3)	28	0	0	0	1,114	0	0	1 (0.1)
VSD (type unknown)	0	0	0	0	0	0	0	0	1	0	0	0	142	4 (2.8)	0	4 (2.8)	143	4 (2.8)	0	4 (2.8)
VSD + PS	1	0	0	0	37	0	0	0	32	0	0	0	2	0	0	0	72	0	0	0
DCRV ± VSD	0	0	0	0	6	0	0	0	28	0	0	0	4	0	0	0	38	0	0	0
Aneurysm of sinus of Valsalva	0	0	0	0	0	0	0	0	0	0	0	0	5	0	0	0	5	0	0	0
TOF	9	0	0	0	189	3 (1.6)	0	4 (2.1)	227	1 (0.4)	0	1 (0.4)	38	0	0	0	463	4 (0.9)	0	5 (1.1)
PA + VSD	7	0	1 (14.3)	0	80	4 (5.0)	0	5 (6.3)	136	0	0	0	11	0	0	0	234	4 (1.7)	1 (0.4)	5 (2.1)
DORV	26	2 (7.7)	0	3 (11.5)	148	2 (1.4)	0	4 (2.7)	154	0	0	1 (0.6)	11	0	0	0	339	4 (1.2)	0	8 (2.4)
TGA (simple)	86	2 (2.3)	0	2 (2.3)	4	0	0	0	2	0	0	0	2	0	0	0	94	2 (2.1)	0	2 (2.1)
+ VSD	42	1 (2.4)	0	3 (7.1)	18	0	0	2 (11.1)	10	0	0	0	2	0	0	0	72	1 (1.4)	0	5 (6.9)
VSD + PS	0	0	0	0	1	0	0	0	0	0	0	0	1	0	0	0	2	0	0	0
Corrected TGA	0	0	0	0	8	0	0	0	25	0	0	0	10	0	0	1 (10.0)	43	0	0	1 (2.3)
Truncus arteriosus	7	0	0	0	25	0	0	1 (4.0)	22	0	0	0	1	0	0	0	55	0	0	1 (1.8)
SV	17	2 (11.8)	0	4 (23.5)	138	2 (1.4)	0	7 (5.1)	179	3 (1.7)	1 (0.6)	6 (3.4)	22	1 (4.5)	0	1 (4.5)	356	8 (2.2)	1 (0.3)	18 (5.1)
TA	3	0	0	0	47	1 (2.1)	0	3 (6.4)	52	0	0	1 (1.9)	10	0	0	0	112	1 (0.9)	0	4 (3.6)
HLHS	35	4 (11.4)	0	11 (31.4)	113	2 (1.8)	0	6 (5.3)	78	3 (3.8)	0	3 (3.8)	0	0	0	0	226	9 (4.0)	0	20 (8.8)
Aortic valve lesion	3	0	0	0	27	0	0	1 (3.7)	115	0	0	1 (0.9)	42	1 (2.4)	0	1 (2.4)	187	1 (0.5)	0	3 (1.6)
Mitral valve lesion	1	0	0	0	31	1 (3.2)	0	3 (9.7)	83	0	0	0	20	0	0	1 (5.0)	135	1 (0.7)	0	4 (3.0)
Ebstein	10	1 (10.0)	0	1 (10.0)	17	0	0	0	30	0	0	0	14	0	0	1 (7.1)	71	1 (1.4)	0	2 (2.8)
Coronary disease	1	0	0	0	17	0	0	2 (11.8)	25	0	0	0	4	0	0	0	47	0	0	2 (4.3)
Others	9	1 (11.1)	0	1 (11.1)	32	1 (3.1)	0	2 (6.3)	43	1 (2.3)	0	3 (7.0)	237	3 (1.3)	0	3 (1.3)	321	6 (1.9)	0	9 (2.8)
Conduit failure	1	0	0	0	0	0	0	0	20	0	0	0	6	0	0	0	27	0	0	0
Redo (excluding conduit failure)	1	0	0	0	52	1 (1.9)	0	2 (3.8)	78	0	0	1 (1.3)	68	1 (1.5)	0	3 (4.4)	199	2 (1.0)	0	6 (3.0)
Total	508	21 (4.1)	1 (0.2)	42 (8.3)	2257	24 (1.1)	0	54 (2.4)	2772	9 (0.3)	1 (0.0)	23 (0.8)	1593	24 (1.5)	0	29 (1.8)	7130	78 (1.1)	2 (0.0)	148 (2.1)
( ), % mortality																				
*CPB* cardiopulmonary bypass, *PDA* patent ductus arteriosus, *VSD* ventricular septal defect, *DORV* double-outlet right ventricle, *AVSD* atrioventricular septal defect, *TGA* transposition of great arteries, *SV* single ventricle, *Interrupt. of Ao.* interruption of aorta, *PS* pulmonary stenosis, *PA-IVS* pulmonary atresia with intact ventricular septum, *TAPVR* total anomalous pulmonary venous return, *PAPVR* partial anomalous pulmonary venous return, *ASD* atrial septal defect, *TOF* tetralogy of Fallot, *DCRV* double-chambered right ventricle, *TA* tricuspid atresia, *HLHS* hypoplastic left heart syndrome, *RV-PA* right ventricle-pulmonary artery																				

(2) CPB ( −) (total; 2123)																					
	Neonate				Infant					1–17 years				≥ 18 years				Total			
	Cases	30-Day mortality		Hospital mortality	Cases	30-Day mortality		Hospital mortality		Cases	30-Day mortality		Hospital mortality	Cases	30-day mortality		Hospital mortality	Cases	30-Day mortality		Hospital mortality
		Hospital	After discharge			Hospital	After discharge				Hospital	After discharge			Hospital	After discharge			Hospital	After discharge	
PDA	305	5 (1.6)	0	12 (3.9)	151	2 (1.3)	0	5 (3.3)		25	0	0	0	2	0	0	0	483	7 (1.4)	0	17 (3.5)
Coarctation (simple)	22	0	0	0	15	0	0	0		1	0	0	0	1	0	0	0	39	0	0	0
+ VSD	47	0	0	0	14	0	0	1 (7.1)		1	0	0	0	0	0	0	0	62	0	0	1 (1.6)
+ DORV	3	0	0	0	0	0	0	0		1	0	0	0	0	0	0	0	4	0	0	0
+ AVSD	4	0	0	2 (50.0)	0	0	0	0		0	0	0	0	0	0	0	0	4	0	0	2 (50.0)
+ TGA	0	0	0	0	1	0	0	0		0	0	0	0	0	0	0	0	1	0	0	0
+ SV	0	0	0	0	0	0	0	0		0	0	0	0	0	0	0	0	0	0	0	0
+ Others	8	0	0	0	5	0	0	0		1	0	0	0	0	0	0	0	14	0	0	0
Interrupt. of Ao (simple)	0	0	0	0	0	0	0	0		0	0	0	0	0	0	0	0	0	0	0	0
+ VSD	29	1 (3.4)	0	2 (6.9)	8	0	0	0		2	0	0	0	0	0	0	0	39	1 (2.6)	0	2 (5.1)
+ DORV	0	0	0	0	0	0	0	0		0	0	0	0	0	0	0	0	0	0	0	0
+ Truncus	1	0	0	0	0	0	0	0		0	0	0	0	0	0	0	0	1	0	0	0
+ TGA	0	0	0	0	0	0	0	0		0	0	0	0	0	0	0	0	0	0	0	0
+ Others	2	0	0	0	0	0	0	0		0	0	0	0	0	0	0	0	2	0	0	0
Vascular ring	5	0	0	0	15	0	0	1 (6.7)		6	0	0	0	0	0	0	0	26	0	0	1 (3.8)
PS	4	0	0	0	7	0	0	0		1	0	0	0	0	0	0	0	12	0	0	0
PA∙IVS or Critical PS	15	0	0	0	20	0	0	0		3	0	0	1 (33.3)	0	0	0	0	38	0	0	1 (2.6)
TAPVR	16	1 (6.3)	0	1 (6.3)	12	0	0	0		3	0	0	0	0	0	0	0	31	1 (3.2)	0	1 (3.2)
PAPVR ± ASD	0	0	0	0	0	0	0	0		1	0	0	0	1	0	0	0	2	0	0	0
ASD	2	0	0	0	2	0	0	0		1	0	0	0	3	0	0	0	8	0	0	0
Cor triatriatum	0	0	0	0	1	0	0	0		0	0	0	0	0	0	0	0	1	0	0	0
AVSD (partial)	1	0	0	0	2	0	0	0		0	0	0	0	0	0	0	0	3	0	0	0
AVSD (complete)	50	0	0	2 (4.0)	70	0	0	1 (1.4)		13	0	0	0	1	0	0	0	134	0	0	3 (2.2)
+ TOF or DORV	3	0	0	0	6	0	0	0		2	0	0	0	1	0	0	0	12	0	0	0
+ Others	0	0	0	0	0	0	0	0		0	0	0	0	0	0	0	0	0	0	0	0
VSD (subarterial)	1	0	0	0	6	0	0	0		0	0	0	0	0	0	0	0	7	0	0	0
VSD (perimemb./muscular)	46	2 (4.3)	0	4 (8.7)	119	0	0	1 (0.8)		6	0	0	0	1	0	0	0	172	2 (1.2)	0	5 (2.9)
VSD (Type Unknown)	0	0	0	0	0		0	0		0	0	0	0	0	0	0	0	0	0	0	0
VSD + PS	0	0	0	0	2	0	0	0		0	0	0	0	0	0	0	0	2	0	0	0
DCRV ± VSD	0	0	0	0	1	0	0	0		0	0	0	0	0	0	0	0	1	0	0	0
Aneurysm of sinus of Valsalva	0	0	0	0	0	0	0	0		0	0	0	0	0	0	0	0	0	0	0	0
TOF	19	0	0	0	82	0	0	0		11	0	0	0	2	0	0	0	114	0	0	0
PA + VSD	15	1 (6.7)	0	1 (6.7)	38	0	0	0		20	0	1 (5.0)	0	1	0	0	0	74	1 (1.4)	1 (1.4)	1 (1.4)
DORV	45	0	0	1 (2.2)	74	1 (1.4)	1 (1.4)	2 (2.7)		12	0	0	0	1	0	0	0	132	1 (0.8)	1 (0.8)	3 (2.3)
TGA (simple)	4	0	0	0	2	0	0	0		0	0	0	0	0	0	0	0	6	0	0	0
+ VSD	13	0	0	0	6	0	0	0		0	0	0	0	1	0	0	0	20	0	0	0
VSD + PS	0	0	0	0	0	0	0	0		0	0	0	0	0	0	0	0	0	0	0	0
Corrected TGA	6	0	0	0	8	0	0	0		9	0	0	0	0	0	0	0	23	0	0	0
Truncus arteriosus	18	0	0	1 (5.6)	5	0	0	0		1	0	0	0	0	0	0	0	24	0	0	1 (4.2)
SV	48	2 (4.2)	0	3 (6.3)	40	1 (2.5)	0	1 (2.5)		20	0	0	1 (5.0)	2	0	0	0	110	3 (2.7)	0	5 (4.5)
TA	14	0	0	0	19	0	0	0		3	0	0	0	4	0	0	0	40	0	0	0
HLHS	80	1 (1.3)	0	3 (3.8)	28	0	0	2 (7.1)		7	0	0	0	0	0	0	0	115	1 (0.9)	0	5 (4.3)
Aortic valve lesion	5	0	0	0	2	0	0	0		2	0	0	0	1	0	0	0	10	0	0	0
Mitral valve lesion	3	0	0	0	5	0	0	0		4	0	0	0	0	0	0	0	12	0	0	0
Ebstein	6	1 (16.7)	0	1 (16.7)	4	0	0	0		3	0	0	0	0	0	0	0	13	1 (7.7)	0	1 (7.7)
Coronary disease	0	0	0	0	8	0	0	0		0	0	0	0	1	0	0	0	9	0	0	0
Others	9	0	0	2 (22.2)	13	2 (15.4)	0	3 (23.1)		18	3 (16.7)	0	3 (16.7)	4	0	0	0	44	5 (11.4)	0	8 (18.2)
Conduit failure	0	0	0	0	1	0	0	0		1	0	0	0	1	0	0	0	3	0	0	0
Redo (excluding conduit failure)	26	0	0	0	113	2 (1.8)	0	5 (4.4)		110	0	0	0	27	0	0	0	276	2 (0.7)	0	5 (1.8)
Total	875	14 (1.6)	0	35 (4.0)	905	8 (0.9)	1 (0.1)	22 (2.4)		288	3 (1.0)	1 (0.3)	5 (1.7)	55	0	0	0	2,123	25 (1.2)	2 (0.09)	62 (2.9)
( ), % mortality																					
*CPB* cardiopulmonary bypass, *PDA* patent ductus arteriosus, *VSD* ventricular septal defect, *DORV* double-outlet right ventricle, *AVSD* atrioventricular septal defect, *TGA* transposition of the great arteries, *SV* single ventricle, *Interrupt. of Ao*. interruption of aorta, *PS* pulmonary stenosis; PA-IVS, pulmonary atresia with intact ventricular septum; TAPVR, total anomalous pulmonary venous return; PAPVR, partial anomalous pulmonary venous return, *ASD* atrial septal defect, *TOF* tetralogy of Fallot, *DCRV* double-chambered right ventricle, *TA* tricuspid atresia, *HLHS* hypoplastic left heart syndrome, *RV-PA* right ventricle-pulmonary artery																					

(3) Main procedure																					
		Neonate				Infant				1–17 years				≥ 18 years				Total			
		Cases	30-Day mortality		Hospital mortality	Cases	30-Day mortality		Hospital mortality	Cases	30-Day mortality		Hospital mortality	Cases	30-Day mortality		Hospital mortality	Cases	30-Day mortality		Hospital mortality
				After discharge			Hospital	After discharge			Hospital	After discharge			Hospital	After discharge			Hospital	After discharge	
1	SP Shunt	102	2 (2.0)	0	3 (2.9)	360	6 1.7)	1 (0.3)	10 (2.8)	50	0	0	4 (8.0)	1	0	0	0	513	8 (1.6)	1 (0.2)	17 (3.3)
2	PAB	275	2 (0.7)	0	9 (3.3)	304	0	0	5 (1.6)	20	0	0	0	2	0	0	0	601	2 (0.3)	0	14 (2.3)
3	Bidirectional Glenn or hemi-Fontan ± α	1	0	0	0	257	1 (0.4)	0	5 (1.9)	98	2 (2.0)	0	3 (3.1)	3	0	0	0	359	3 (0.8)	0	8 (2.2)
4	Damus-Kaye-Stansel operation	0	0	0	0	27	0	0	2 (7.4)	8	0	0	0	1	0	0	0	36	0	0	2 (5.6)
5	PA reconstruction/repair (including redo)	16	0	0	1 (6.3)	180	2 (1.1)	0	4 (2.2)	195	0	0	3 (1.5)	16	0	0	0	407	2 (0.5)	0	8 (2.0)
6	RVOT reconstruction/repair	5	0	1 (20.0)	0	208	2 (1.0)	0	3 (1.4)	309	1 (0.3)	0	1 (0.3)	43	0	0	0	565	3 (0.5)	1 (0.2)	4 (0.7)
7	Rastelli procedure	2	0	0	0	49	1 (2.0)	0	1 (2.0)	114	0	0	1 (0.9)	2	0	0	0	167	1 (0.6)	0	2 (1.2)
8	Arterial switch procedure	140	6 (4.3)	0	9 (6.4)	23	1 (4.3)	0	2 (8.7)	2	0	0	0	0	0	0	0	165	7 (4.2)	0	11 (6.7)
9	Atrial switch procedure	0	0	0	0	0	0	0	0	4	0	0	0	2	0	0	0	6	0	0	0
10	Double switch procedure	0	0	0	0	1	0	0	0	6	0	0	0	0	0	0	0	7	0	0	0
11	Repair of anomalous origin of CA	0	0	0	0	9	0	0	2 (22.2)	4	0	0	0	0	0	0	0	13	0	0	2 (15.4)
12	Closure of coronary AV fistula	1	0	0	0	4	0	0	0	6	0	0	0	2	0	0	0	13	0	0	0
13	Fontan/TCPC	0	0	0	0	1	0	0	0	353	1 (0.3)	0	4 (1.1)	41	2 (4.9)	0	2 (4.9)	395	3 (0.8)	0	6 (1.5)
14	Norwood procedure	31	3 (9.7)	0	5 (16.1)	95	7 (7.4)	0	14 (14.7)	4	1 (25.0)	0	1 (25.0)	0	0	0	0	130	11(8.5)	0	20 (15.4)
15	Ventricular septation	0	0	0	0	0	0	0	0	0	0	0	0	0	0	0	0	0	0	0	0
16	Left side AV valve repair (including Redo)	0	0	0	0	23	0	0	0	88	0	0	0	17	0	0	0	128	0	0	0
17	Left side AV valve replace (including Redo)	1	0	0	0	10	0	0	1 (10.0)	46	0	0	1 (2.2)	18	0	0	2 (11.1)	75	0	0	4 (5.3)
18	Right side AV valve repair (including Redo)	12	2 (16.7)	0	2 (16.7)	71	0	0	0	81	0	0	0	67	0	0	1 (1.5)	231	2 (0.9)	0	3 (1.3)
19	Right side AV valve replace (including Redo)	0	0	0	0	1	0	0	0	9	1 (11.1)	0	1 (11.1)	26	0	0	0	36	1 (2.8)	0	1 (2.8)
20	Common AV valve repair (including Redo)	3	0	0	2 (66.7)	17	0	0	1 (5.9)	11	0	0	0	2	0	0	0	33	0	0	3(9.1)
21	Common AV valve replace (including Redo)	0	0	0	0	4	1 (25.0)	0	2 (50.0)	7	0	1 (14.3)	0	2	0	0	0	13	1 (7.7)	1 (7.7)	2 (15.4)
22	Repair of supra-aortic stenosis	0	0	0	0	5	0	0	1 (20.0)	16	0	0	0	1	0	0	0	22	0	0	1 (4.5)
23	Repair of subaortic stenosis (including Redo)	0	0	0	0	8	0	0	0	42	0	0	0	5	0	0	0	55	0	0	0
24	Aortic valve plasty ± VSD Closure	4	0	0	0	15	0	0	1 (6.7)	29	0	0	0	5	0	0	0	53	0	0	1 (1.9)
25	Aortic valve replacement	0	0	0	0	2	0	0	0	32	0	0	0	30	1 (3.3)	0	1 (3.3)	64	1 (1.6)	0	1 (1.6)
26	AVR with annular enlargement	0	0	0	0	3	0	0	0	9	0	0	1 (11.1)	2	0	0	0	14	0	0	1 (7.1)
27	Aortic root Replace (except Ross)	0	0	0	0	0	0	0	0	8	0	0	0	19	0	0	0	27	0	0	0
28	Ross procedure	0	0	0	0	3	0	0	0	13	0	0	0					16	0	0	0
29	Bilateral pulmonary artery banding	175	5 (2.9)	0	14 (8.0)	8	0	0	1 (12.5)	0	0	0	0	0	0	0	0	183	5 (2.7)	0	15 (8.2)
Total		768	20 (2.6)	1 (0.1)	45 (5.9)	1688	21 (1.2)	1 (0.1)	55 (3.3)	1564	6 (0.4)	1 (0.1)	20 (1.3)	307	3 (1.0)	0	6 (2.0)	4327	50 (1.2)	3 (0.07)	126 (2.9)
( ), % mortality																					
*SP* systemic-pulmonary, *PAB* pulmonary artery banding, *PA* pulmonary artery, *RVOT* right ventricular outflow tract, *CA* coronary artery, *AV* fistula, arteriovenous fistula, *TCPC* total cavopulmonary connection, *AV valve* atrioventricular valve, *VSD* ventricular septal defect, *AVR* aortic valve replacement

**Table 4:** Acquired (total, (1) + (2) + (4) + (5) + (6) + (7) + isolated operations for arrhythmia in (3); 39,307 (1) Valvelar heart disease (total; 23,205)

	Valve	Cases	Operation					30-Day mortality				Hospital mortality		Redo			
			Mechanical	Bioprosthesis	Repair	Unknown	With CABG	Hospital		After discharge				Cases	30-Day mortality		Hospital mortality
								Replace	Repair	Replace	Repair	Replace	Repair		Hosipital	After discharge	
Isolated	A	10,584	1512	8427	261	384	2562	168 (1.7)	2 (0.8)	7 (0.1)	0	295 (3.0)	6 (2.3)	688	33 (4.8)	0	53 (7.7)
	M	4898	479	887	3447	85	577	59 (4.3)	35 (1.0)	0	0	97 (7.1)	50 (1.5)	595	15 (2.5)	0	35 (5.9)
	T	596	8	84	495	9	63	3 (3.3)	9 (1.8)	0	0	8 (8.7)	24 (4.9)	113	3 (2.7)	0	11 (9.7)
	P	22	0	17	5	0	1	0	2 (40)	0	0	0	2 (40)	12	0	0	0
A + M		1326					206	61 (4.6)		0		99 (7.5)		133	4 (3.0)	0	13 (9.8)
	A		276	950	48	52											
	M		186	378	727	35											
A + T		599					95	17(2.8)		0		36 (6.0)		69	4 (5.8)	0	5 (7.3)
	A		68	491	16	24											
	T		1	11	574	13											
M + T		3937					371	74(1.9)		0		128 (3.3)		474	16 (3.4)	0	33 (7.0)
	M		370	1070	2437	60											
	T		1	56	3847	33											
A + M + T		1135					117	49(4.3)		0		72 (6.3)		138	10 (7.3)	0	14 (10.0)
	A		200	876	23	36											
	M		147	422	536	30											
	T		0	10	1116	9											
Others		108					15	0		0		2 (1.9)		22	0	0	1 (4.6)
Total		23,205					4007	479(2.1)		7 (0.03)		819 (3.5)		2244	85 (3.7)	0	165 (7.4)
*A* aortic valve, *M* mitral valve, *T* tricuspid valve, *P* pulmonary valve																	
*CABG* coronary artery bypass grafting																	

TAVR	Cases	30-Day mortality	
	6610	69	(1.0)
*TAVR* transcatheter aortic valve replacement			

(2) Ischemic heart disease (total, (A) + (B); 13,445)																					
(A) Isolated CABG (total; (a) + (b); 12,135)																					
(a-1) On-pump arrest CABG (total; 2662)																					
	Primary, elective				Primary, emergent				Redo, elective				Redo, emergent				Artery only	Artery + svg	Svg only	Others	Unclear
	Cases	30 Day mortality		Hospital mortality	Cases	30 Day mortality		Hospital mortality	Cases	30 Day mortality		Hospital mortality	Cases	30 Day mortality		Hospital mortality					
		Hospital	After discharge			Hospital	After discharge			Hospital	After discharge			Hospital	After discharge						
1VD	56	1 (1.8)	0	1	5	0	0	0	0	0	0	0	2	0	0	0	23	27	11	1	1
2VD	292	0	0	3 (1.0)	35	2 (5.7)	0	4 (11.4)	2	0	0	0	0	0	0	0	38	273	16	0	2
3VD	985	11 (1.1)	0	19 (1.9)	129	10 (7.8)	0	15 (11.6)	1	0	0	0	0	0	0	0	52	1010	41	7	5
LMT	832	8 (1.0)	0	14 (1.7)	226	14 (6.2)	0	17 (7.5)	8	0	0	0	3	0	0	1 (33.3)	86	916	60	2	5
No info	70	0	0	0	15	1 (6.7)	0	1 (6.7)	0	0	0	0	1	0	0	1 (100.0)	23	48	9	2	3
Total	2235	20 (0.9)	0	37 (1.7)	410	27 (6.6)	0	37 (9.0)	11	0	0	0	6	0	0	2 (33.3)	222	2275	137	12	16
Kawasaki	4	0	0	0	1	0 (0.0)	0	0	0	0	0	0	0	0	0	0	2	2	1	0	0
On dialysis	251	5 (2.0)	0	11 (4.4)	41	8 (19.5)	0	12 (29.3)	4	0	0	0	3	1 (33.3)	0	1 (33.3)	9	262	22	0	3
( ), % mortality																					
LMT includes LMT alone or LMT with other branch diseases																					
*CABG* coronary artery bypass grafting, *1VD* one-vessel disease, *2VD* two-vessel disease, *3VD* three-vessel disease, *LMT* left main trunk, *SVG* saphenous vein graft																					

(a-2) On-pump beating CABG (total; 2276)																					
	Primary, elective				Primary, emergent				Redo, elective				Redo, emergent				Artery only	Artery + svg	Svg only	Others	Unclear
	Cases	30 Day mortality		Hospital mortality	Cases	30 Day mortality		Hospital mortality	Cases	30 day mortality		Hospital mortality	Cases	30 Day mortality		Hospital mortality					
		Hospital	After discharge			Hospital	After discharge			Hospital	After discharge			Hospital	After discharge						
1VD	27	0	0	0 (0.0)	11	1 (9.1)	0	1 (9.1)	2	0	0	0	0	0	0	0	13	21	6	0	0
2VD	199	6 (3.0)	0	11 (5.5)	41	5 (12.2)	0	8 (19.5)	5	0	0	0	1	0	0	0	45	174	17	1	9
3VD	686	5 (0.7)	0	13 (1.9)	193	15 (7.8)	0	21 (10.9)	4	0	0	1 (25.0)	0	0	0	0	73	760	40	6	4
LMT	669	7 (1.0)	0	14 (2.1)	341	26 (7.6)	1 (0.3)	39 (11.4)	17	1 (5.9)	0	1 (5.9)	2	0	0	0	157	798	65	2	7
no info	49	0 (0.0)	0	0 (0.0)	26	1 (3.8)	0	3 (11.5)	1	0	0	0	2	0	0	0	11	55	12	0	0
Total	1630	18 (1.1)	0	38 (2.3)	612	48 (7.8)	1 (0.2)	72 (11.8)	29	1 (3.4)	0	2 (6.9)	5	0	0	0	299	1808	140	9	20
Kawasaki	3	0	0	0	0	0	0	0	0	0	0	0	0	0	0	0	0	3	0	0	0
On dialysis	221	11 (5.0)	0	23 (10.4)	87	11(12.6)	1 (1.1)	13 (14.9)	6	1 (16.7)	0	2	1	0	0	0	34	244	30	2	5
(), % mortality																					
LMT includes LMT alone or LMT with other branch diseases																					
*CABG* coronary artery bypass grafting, *1VD* one-vessel disease, *2VD* two-vessel disease, *3VD* three-vessel disease, *LMT* left main trunk, *SVG* saphenous vein graft																					

(b) Off-pump CABG (total; 7197)																					
(Including cases of planned off-pump CABG in which, during surgery, the change is made to an on-pump CABG or on-pump beating-heart procedure)																					
	Primary, elective				Primary, emergent				Redo, elective				Redo, emergent				Artery only	Artery + svg	Svg only	Others	Unclear
	Cases	30 Day mortality		Hospital mortality	Cases	30 Day mortality		Hospital mortality	Cases	30 Day mortality		Hospital mortality	Cases	30 Day mortality		Hospital mortality					
		Hospital	After discharge			Hospital	After discharge			Hospital	After discharge			Hospital	After discharge						
1VD	314	3 (1.0)	0	4 (1.3)	43	0	0	1 (2.3)	8	0	0	0	1	0	0	0	248	77	38	1	2
2VD	935	6 (0.6)	0	8 (0.9)	129	1 (0.8)	0	3 (2.3)	9	0	0	0	1	0	0	0	373	649	44	4	4
3VD	2401	21 (0.9)	0	38(1.6)	317	14 (4.4)	0	19 (6.0)	13	0	0	1 (7.7)	1	0	0	0	569	2085	53	15	10
LMT	2252	6 (0.3)	2(0.1)	14 (0.6)	525	19 (3.6)	0	25 (4.8)	18	0	0	2	5	1 (20.0)	0	1 (20.0)	761	1929	86	7	17
No info	175	1 (0.6)	1 (0.6)	1 (0.6)	41	0	0	2 (4.9)	8	0	0	0	1	0	0	0	82	132	8	2	1
Total	6077	37 (0.6)	3 (0.0)	65 (1.1)	1055	34 (3.2)	0	50 (4.7)	56	0	0	3 (5.4)	9	1 (11.1)	0	1 (11.1)	2033	4872	229	29	34
Kawasaki	15	0	0	0	3	0	0	0	1	0	0	0	0	0	0	0	12	6	1	0	0
On dialysis	738	14 (1.9)	1 (0.1)	31 (4.2)	127	7 (5.5)	0	11 (8.7)	13	0	0	2 (15.4)	2	1 (50.0)	0	1 (50.0)	205	627	38	4	5
(), % mortality																					
LMT includes LMT alone or LMT with other branch diseases																					
*CABG* coronary artery bypass grafting, *1VD* one-vessel disease, *2VD* two-vessel disease, *3VD* three-vessel disease, *LMT* left main trunk, *SVG* saphenous vein graft																					

(c) Cases of conversion, during surgery, from off-pump CABG to on-pump CABG or on- pump beating-heart CABG [these cases are also included in category (b)]																
	Primary, elective				Primary, emergent				Redo, elective				Redo, emergent			
	Cases	30 Day mortality		Hospital mortality	Cases	30 Day mortality		Hospital mortality	Cases	30 Day mortality		Hospital mortality	Cases	30 Day mortality		Hospital mortality
		Hospital	After discharge			Hospital	After discharge			Hospital	After discharge			Hospital	After discharge	
Converted to arrest	30	0	0	2 (6.7)	4	0	0	0	1	0	0	0	0	0	0	0
Converted to beating	120	2 (1.7)	0	3 (2.5)	33	5 (15.2)	0	6 (18.2)	1	0	0	0	0	0	0	0
Total	150	2 (1.3)	0	5 (3.3)	37	5 (13.5)	0	6 (16.2)	2	0	0	0	0	0	0	0
On dialysis	24	2 (8.3)	0	5 (20.8)	9	3 (33.3)	0	4 (44.4)	1	0	0	0	0	0	0	0
( ), % mortality																
*CABG* coronary artery bypass grafting																

(B) Operation for complications of MI (total; 1310)											
	Chronic				Acute				Concomitant operation		
	Cases	30-Day mortality		Hospital mortality	Cases	30-Day mortality		Hospital mortality			
		Hospital	After discharge			Hospital	After discharge		CABG	MVP	MVR
Infarctectomy or Aneurysmectomy	108	6 (5.6)	0	7 (6.5)	26	5 (19.2)	0	8 (30.8)	77	24	13
VSP closure	78	12 (15.4)	0	20 (25.6)	284	68 (23.9)	0	112 (39.4)	95	3	3
Cardiac rupture	25	5 (20.0)	0	5 (20.0)	227	72 (31.7)	0	87 (38.3)	32	4	2
Mitral regurgitation											
(1) Papillary muscle rupture	8	1 (12.5)	0	1 (12.5)	58	22 (37.9)	0	23 (39.7)	29	11	55
(2) Ischemic	250	10 (4.0)	0	18 (7.2)	50	9 (18.0)	0	10 (20.0)	221	169	131
Others	89	4 (4.5)	0	7 (7.9)	107	23 (21.5)	0	36 (33.6)	68	13	6
Total	558	38 (6.8)	0	58 (10.4)	752	199 (26.5)	0	276 (36.7)	522	224	210
( ), % mortality											
Acute, within 2 weeks from the onset of myocardial infarction											
*MI* myocardial infarction, *CABG* coronary artery bypass grafting, *MVP* mitral valve repair, *MVR* mitral valve replacement, *VSP* ventricular septal perforation											

(3) Operation for arrhythmia (total; 5334)											
	Cases	30-Day mortality		Hospital mortality	Concomitant operation						
					Isolated	Congenital	Valve	IHD	Others	Multiple combination	
		Hospital	After discharge							2 Categories	3 Categories
Maze	3274	64 (2.0)	2 (0.06)	106 (3.2)	136	177	2792	540	292	637	43
For WPW	3	0	0	0	0	0	1	2	0	0	0
For ventricular tachyarrhythmia	33	1 (3.0)	0	1 (3.0)	3	0	15	18	3	0	0
Others	2024	39 (1.9)	0	65 (3.2)	31	113	1708	359	200	397	32
Total	5334	104 (1.9)	2 (0.04)	172 (3.2)	170	290	4516	919	495	1034	75
( ), % mortality											
Except for 170 isolated cases, all remaining 5,164 cases are doubly allocated, one for this subgroup and the other for the subgroup corresponding to the concomitant operations.											
*WPW* Wolff–Parkinson–White syndrome,* IHD* ischemic heart disease											

(4) Operation for constrictive pericarditis (total; 210)								
	CPB (+)				CPB (−)			
	Cases	30-Day mortality		Hospital mortality	Cases	30-Day mortality		Hospital mortality
		Hospital	After discharge			Hospital	After discharge	
Total	95	4 (4.2)	0	11 (11.6)	115	10 (8.7)	0	17 (14.8)
( ), % mortality								
*CPB* cardiopulmonary bypass								

(5) Cardiac tumor (total; 725)								
	Cases	30-Day mortality		Hospital mortality	Concomitant operation			
		Hospital	After discharge		AVR	MVR	CABG	Others
Benign tumor	625	1 (0.2)	0	9 (1.4)	25	12	45	138
(Cardiac myxoma)	427	5 (1.2)	0	2 (0.5)	10	5	24	79
Malignant tumor	100	3 (3.0)	0	5 (5.0)	1	5	5	22
(Primary)	9	0	0	0	0	1	1	2
( ), % mortality								
*AVR* aortic valve replacement, *MVR* mitral valve replacement, *CABG* coronary artery bypass grafting								

(6) HOCM and DCM (total; 338)								
	Cases	30-Day mortality		Hospital mortality	Concomitant operation			
		Hospital	After discharge		AVR	MVR	MVP	CABG
Myectomy	148	5 (3.4)	0	6 (4.1)	61	17	24	14
Myotomy	12	0	0	1 (8.3)	2	1	3	2
No-resection	171	8 (4.7)	0	14 (8.2)	27	93	78	20
Volume reduction surgery of the left ventricle	7	0	0	0	1	1	2	0
Total	338	13 (3.8)	0	21 (6.2)	91	112	107	36
( ), % mortality								
*HOCM* hypertrophic obstructive cardiomyopathy, *DCM* dilated cardiomyopathy, *AVR* aortic valve replacement, *MVR* mitral valve replacement, *MVP* mitral valve repair, *CABG* coronary artery bypass grafting								

(7) Other open-heart operation (total; 1214)				
	Cases	30-Day mortality		Hospital mortality
		Hospital	After discharge	
Open-heart operation	497	48 (9.7)	0	63 (12.7)
Non-open-heart operation	717	91 (12.7)	0	142 (19.8)
Total	1214	139 (11.4)	0	205 (16.9)

(), % mortality

**Table 5:** Thoracic aortic aneurysm (total; 21,624) (1) Dissection (total; 10,453)

Stanford type	Acute								Chronic								Concomitant operation					
	A				B				A				B									
Replaced site	Cases	30-Day mortality		Hospital mortality	Cases	30-Day mortality		Hospital mortality	Cases	30-Day mortality		Hospital mortality	Cases	30-Day mortality		Hospital mortality	AVP	AVR	MVP	MVR	CABG	Others
		Hospital	After discharge			Hospital	After discharge			Hospital	After discharge			Hospital	After discharge							
Ascending Ao	2354	169 (7.2)	1 (0.04)	202 (8.6)	3	1 (33.3)	0	1 (33.3)	231	7 (3.0)	0	13 (5.6)	6	0	0	0	106	137	18	12	134	116
Aortic Root	238	35 (14.7)	0	42 (17.6)	0	0	0	0	81	7 (8.6)	1 (1.2)	8 (9.9)	5	0	0	0	45	200	5	5	73	16
Arch	1956	151 (7.7)	3 (0.15)	198 (10.1)	32	3 (9.4)	0	4 (12.5)	410	8 (2.0)	0	10 (2.4)	177	5 (2.8)	0	6 (3.4)	84	113	9	7	123	76
Aortic root + asc. Ao. + Arch	186	23 (12.4)	0	26 (14.0)	0	0	0	0	39	2 (5.1)	0	2 (5.1)	7	1 (14.3)	0	1 (14.3)	27	135	4	0	46	8
Descending Ao	60	9 (15.0)	0	9 (15.0)	30	3 (10.0)	0	4 (13.3)	73	2 (2.7)	0	3 (4.1)	262	11 (4.2)	2 (0.8)	12 (4.6)	1	7	0	0	7	3
Thoracoabdominal	15	2 (13.3)	0	2 (13.3)	13	2 (15.4)	0	2 (15.4)	55	4 (7.3)	0	4 (7.3)	212	9 (4.2)	0	16 (7.5)	1	0	0	0	3	2
Simple TEVAR	68	9 (13.2)	0	9 (13.2)	327	16 (4.9)	1 (0.3)	23 (7.0)	205	1 (0.5)	0	2 (1.0)	1032	15 (1.5)	2 (0.2)	21 (2.0)	0	1	0	0	1	5
Open SG with BR	873	86 (9.9)	1 (0.11)	102 (11.7)	35	3 (8.6)	0	4 (11.4)	187	10 (5.3)	0	13 (7.0)	212	3 (1.4)	1 (0.5)	6 (2.8)	38	106	6	2	83	29
Open SG without BR	362	33 (9.1)	0	44 (12.2)	25	4 (16.0)	0	4 (16.0)	47	1 (2.1)	0	4 (8.5)	82	2 (2.4)	0	3 (3.7)	34	37	1	0	26	9
Arch TEVAR with BR	18	0	0	0	85	0	0	2 (2.4)	47	0	0	0	298	2 (0.7)	1 (0.3)	5 (1.7)	0	0	0	0	0	13
Thoracoabdominal TEVAR with BR	6	2 (33.3)	0	2 (33.3)	15	0	0	0	7	0	0	0	43	3 (7.0)	0	5 (11.6)	0	1	0	0	1	2
Other	21	9 (42.9)	0	10 (47.6)	3	1 (33.3)	0	1 (33.3)	2	0	0	0	8	1 (12.5)	0	1 (12.5)	0	0	0	0	1	3
Total	6157	398 (6.5)	5 (0.08)	646 (10.5)	568	33 (5.8)	1 (0.2)	45 (7.9)	1384	42 (3.0)	1 (0.1)	59 (4.3)	2344	52 (2.2)	6 (0.3)	76 (3.2)	336	737	43	26	498	282
(), % mortality																						
*Ao* aorta, *AVP* aortic valve repair, *AVR* aortic valve replacement, *MVP* mitral valve repair, *MVR* mitral valve replacement, *CABG* coronary artery bypass grafting, *TEVAR* thoracic endovascular aortic (aneurysm) repair, *SG* stentgraft, *BR* branch reconstruction																						
Acute, within 2 weeks from the onset																						

(2) Non-dissection (total; 11,171)														
Replaced site	Unruptured				Ruptured				Concomitant operation					
	Cases	30-Day mortality		Hospital mortality	Cases	30-Day mortality		Hospital mortality	AVP	AVR	MVP	MVR	CABG	Others
		Hospital	After discharge			Hospital	After discharge							
Ascending Ao	1366	29 (2.1)	0	45 (3.3)	53	11 (20.8)	0	12 (22.6)	68	974	111	48	183	252
Aortic Root	1125	31 (2.8)	0	41 (3.6)	47	9 (19.1)	0	11 (23.4)	276	803	71	30	158	156
Arch	2198	43 (2.0)	0	67 (3.0)	119	19 (16.0)	1 (0.84)	26 (21.8)	37	560	37	25	338	195
Aortic root + asc. Ao. + Arch	275	11 (4.0)	0	17 (6.2)	3	0	0	1 (33.3)	63	185	8	3	31	29
Descending Ao	294	6 (2.0)	0	10 (3.4)	53	9 (17.0)	0	13 (24.5)	1	8	2	0	14	9
Thoracoabdominal	387	30 (7.8)	0	44 (11.4)	38	5 (13.2)	1 (2.63)	8 (21.1)	1	0	0	0	1	1
Simple TEVAR	2143	24 (1.1)	0	44 (2.1)	318	37 (11.6)	0	61 (19.2)	0	2	1	0	0	18
Open SG with BR	1004	20 (2.0)	0	49 (4.9)	69	16 (23.2)	0	22 (31.9)	9	93	8	2	161	69
Open SG without BR	339	10 (2.9)	0	18 (5.3)	34	6 (17.6)	0	9 (26.5)	11	45	2	0	47	23
Arch TEVAR with BR	1004	28 (2.8)	0	40 (4.0)	75	12 (16.0)	0	17 (22.7)	0	2	0	0	5	27
Thoracoabdominal TEVAR with BR	86	4 (4.7)	0	4 (4.7)	15	3 (20.0)	0	4 (26.7)	0	1	0	0	0	0
Other	106	5 (4.7)	0	7 (6.6)	20	5 (25.0)	0	6 (30.0)	1	25	6	2	14	16
Total	10,327	241 (2.3)	0	386 (3.7)	844	132 (15.6)	2 (0.24)	190 (22.5)	467	2698	246	110	952	795
(), % mortality														
*Ao* aorta, *AVP* aortic valve repair, *AVR* aortic valve replacement, *MVP* mitral valve repair, *MVR* mitral valve replacement, *CABG* coronary artery bypass grafting, *TEVAR* thoracic endovascular aortic (aneurysm) repair, *SG* stentgraft, *BR* branch reconstruction

**Table 6:** Pulmonary thromboembolism (total; 138)

	Cases	30-Day mortality		Hospital mortality
		Hospital	After discharge	
Acute	90	10 (11.1)		11
Chronic	48	2 (4.2)		3
Total	138	12 (8.7)	0	14 (10.1)

(), % mortality

**Table 7:** Implantation of VAD (total; 164)

	Cases	30-Day mortality		Hospital mortality
		Hospital	After discharge	
Implantation of VAD	164	3 (1.8)	3 (1.8)	31 (18.9)

(), % mortality

*VAD* ventricular assist devise

**Table 8:** Heart transplantation (total; 51)

	Cases	30-Day mortality		Hospital mortality
		Hospital	After discharge	
Heart Transplantation	51	1 (2.0)	0	2 (3.9)
Heart and Lung Transplantation	0	0	0	0
Total	51	1 (2.0)	0	2 (3.9)

(), % mortality

Among the 9253 procedures for congenital heart disease conducted in 2018, 7130 were open-heart surgeries, with an overall hospital mortality rate of 2.1%. The number of surgeries for neonates and infants in 2018 did not differ significantly compared to that in 2008; however, hospital mortality improved from 10.8 to 8.3% for neonates and from 3.8 to 2.4% for infants. In 2018, atrial septal defect was the most common disease (1402 cases), with patients aged 18 or older accounting for 58.6% of atrial septal defect surgery. Ventricular septal defect (perimembranous/muscular), which had been the most common disease in 2015 and 2016, was the second most common disease (1114 cases).

Within the past 10 years, hospital mortality for complex congenital heart disease was as follows (2008 [2], 2013 [3], and 2018): complete atrio-ventricular septal defect (3.5%, 0.6%, and 2.5%, respectively); tetralogy of Fallot (1.8%, 1.4%, and 1.1%, respectively); transposition of the great arteries with intact septum (3.8%, 3.6%, and 2.1%, respectively), ventricular septal defect (5.5%, 5.2%, and 6.9%, respectively), and single ventricle (5.5%, 5.7%, and 5.1%, respectively); and hypoplastic left heart syndrome (12.9%, 9.1%, and 8.8%, respectively). Currently, right heart bypass surgery has been commonly performed (359 bidirectional Glenn procedures excluding 36 Damus–Kaye–Stansel procedures and 395 Fontan-type procedures including total cavopulmonary connection) with acceptable hospital mortality rates (2.2% and 1.5%). The Norwood type I procedure was performed in 130 cases, with a relatively low hospital mortality rate (15.4%).

The total number of valvular heart disease procedures, excluding transcatheter procedures, was slightly lower than that in the previous year. Moreover, the number of isolated aortic valve replacement/repair with/without coronary artery bypass grafting (CABG) (n = 10,584) was 1.0% lower than that in the previous year (n = 10,690) but 2.0% higher than that 5 years ago (n = 10,379), despite the rapid utilization of transcatheter aortic valve replacement (n = 6610 in 2018). The number of isolated mitral valve replacement/repair with/without CABG (n = 4898) was 4.5% higher than that in the previous year (n = 4687) and 2.2% higher than that 5 years ago (n = 4793). A total of 10,744 and 2757 cases underwent aortic and mitral valve replacement with bioprosthesis, respectively. The rate at which bioprosthesis was utilized had increased dramatically from 30% in the early 2000s [4, 5] to 83.9% and 70.0% in 2018 for aortic and mitral positions, respectively. Additionally, CABG was performed as a concomitant procedure in 17.3% of all valvular procedures (16.7% in 2008 [2] and 17.8% in 2013 [3]). Valve repair had been popular for mitral and tricuspid valve positions (7147 and 6032 cases, respectively), but had been less frequently observed for aortic valve positions (348 patients, only 2.6% of all aortic valve procedures). Mitral valve repair constituted 63.3% of all mitral valve procedures. Hospital mortality rates for single valve replacement were 3.0% and 7.1% for aortic and mitral positions, respectively, but only 1.5% for mitral valve repair. Moreover, hospital mortality rates for redo valve surgery were 7.7% and 5.9% for the aortic and mitral positions, respectively. Finally, overall hospital mortality rates did not improve over the past 10 years (3.3% in 2008 [2], 3.1% in 2013 [3], and 3.5% in 2018).

Isolated CABG had been performed in 12,135 cases, accounting for only 68.3% of the number performed 10 years ago (n = 17,764) [2]. Among the aforementioned cases, 7197 (58.8%) underwent off-pump CABG, with a success rate of 97.4%. The percentage of intended off-pump CABG in 2018 was similar to that in 2017 when it fell below 60% for the first time since 2004 [4]. Hospital mortality associated with primary elective CABG procedures among 7707 cases was 1.3%, which did not differ from that in 2008 (1.5%) [2]. Nonetheless, hospital mortality for primary emergency CABG among 1667 cases still remained high (7.3%). The percentage of conversion from off-pump to on-pump CABG or on-pump beating-heart CABG was 2.6%, with a hospital mortality rate of 5.8%. Patients with end-stage renal failure on dialysis had higher hospital mortality rates than overall mortality, regardless of surgical procedure (on-pump arrest, on-pump beating, and off-pump). In this report, concomitant CABGs alongside other major procedures were not included under the ischemic heart disease category but rather under other categories, such as valvular heart disease and thoracic aortic aneurysm. Accordingly, the overall number of CABGs in 2018, including concomitant CABG with other major procedures, was 17,678.

Measures for arrhythmia were performed primarily as concomitant procedures in 5334 cases, with a hospital mortality rate of 3.2%. Pacemaker and implantable cardioverter–defibrillator implantation was not included in this category.

In 2018, 21,624 procedures for thoracic and thoracoabdominal aortae diseases were performed, among which 10,453 and 11,171 were for aortic dissection and non-dissection, respectively. The number of surgeries for aortic dissection this year was 3.6% higher than that in the preceding year (*n* = 10,086). Hospital mortality rates for the 6157 Stanford type A acute aortic dissections remained high (10.5%). The number of procedures for non-dissected aneurysm increased by 4.8%, with a hospital mortality rate of 5.2% for all aneurysms and 3.7% and 22.5% for unruptured and ruptured aneurysms, respectively. The rate at which thoracic endovascular aortic repair (TEVAR) has been performed for aortic diseases has been increasing. A total of 3974 patients with aortic dissection underwent stent graft placement: 2151 TEVARs and 1823 open stent graftings, respectively. Moreover, 1373 and 294 cases underwent TEVAR and open stent grafting for type B chronic aortic dissection, accounting for 58.6% and 12.5% of the total number of cases, respectively. Hospital mortality rates associated with simple TEVAR for type B aortic dissection were 7.0% and 2.0% for acute and chronic cases, respectively. A total of 5087 patients with non-dissected aortic aneurysm underwent stent graft placement, among which 3641 were TEVARs (a 10.6% increase compared to that in 2017, n = 3292) and 1446 were open stent graftings (a 6.0% increase compared to that in 2017, *n* = 1364). Hospital mortality rates for TEVARs were 2.7% and 20.4% for unruptured and ruptured aneurysms, respectively, whereas those for open stenting were 5.0% and 30.1% for unruptured and ruptured aneurysms, respectively.

### (B) General thoracic surgery

**Fig. 2:**
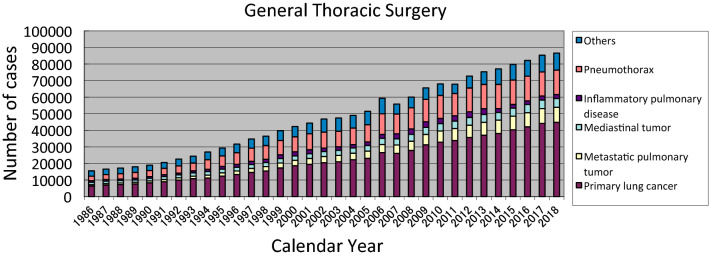
General thoracic surgery

The 2018 survey of general thoracic surgeries comprised 749 surgical units, with the bulk of the data submitted via a web-based collection system established by the NCD [1]. In total, 86,589 procedures had been reported by general thoracic surgery departments in 2018, twice the number of surgeries compared to 2000 and approximately 11,200 more procedures than that in 2013 (Fig. 2).

In 2018, 44,859 procedures for primary lung cancer had been performed, a number that has continued to increase annually. Accordingly, the number of procedures in 2018 was 2.4 times higher than that in 2000, with lung cancer procedures accounting for 52% of all general thoracic surgeries (Table 9).

**Table 9:** Total cases of general thoracic surgery during 2018

	Cases	%
Benign pulmonary tumor	2342	2.7
Primary lung cancer	44,859	51.8
Other primary malignant pulmonary tumor	384	0.4
Metastatic pulmonary tumor	8978	10.4
Tracheal tumor	127	0.1
Mesothelioma	664	0.8
Chest wall tumor	656	0.8
Mediastinal tumor	5361	6.2
Thymectomy for MG without thymoma	151	0.2
Inflammatory pulmonary disease	2400	2.8
Empyema	3103	3.6
Bullous disease excluding pneumothorax	376	0.4
Pneumothorax	14,731	17.0
Chest wall deformity	176	0.2
Diaphragmatic hernia including traumatic	30	0.0
Chest trauma excluding diaphragmatic hernia	431	0.5
Lung transplantation	71	0.1
Others	1749	2.0
Total	86,589	100.0

Information regarding the number of video-assisted thoracoscopic surgery (VATS), defined as surgical procedures utilizing a skin incision over 8 cm and/or a minithoracotomy (hybrid) approach, has been available since the 2015 annual report. The number of VATS procedures for benign pulmonary tumors and primary lung cancer and the total number of VATS procedures in 2016 are presented in Tables 10, 11, 13, 16, 17, 18, 19, 20, 21, 22, 23, 25, 26, 27, respectively.

**Table 10:** Benign pulmonary tumor

	Cases	30-Day mortality		Hospital mortality	By VATS
		Hospital	After discharge		
Benign pulmonary tumor					
Hamartoma	527	0	0	0	503
Sclerosing hemangioma	109	0	0	0	104
Papilloma	23	0	0	0	22
Mucous gland adenoma bronchial	4	0	0	0	4
Fibroma	136	0	0	0	123
Lipoma	8	0	0	0	7
Neurogenic tumor	18	0	0	0	15
Clear cell tumor	2	0	0	0	2
Leiomyoma	12	0	0	0	12
Chondroma	4	0	0	0	4
Inflammatory myofibroblastic tumor	0	0	0	0	0
Pseudolymphoma	26	0	0	0	25
Histiocytosis	12	0	0	0	12
Teratoma	7	0	0	0	6
Others	1454	1 (0.1)	1 (0.1)	1 (0.1)	1383
Total	2342	1 (0.04)	1 (0.04)	1 (0.04)	2222

(), mortality %

**Table 11:** Primary malignant pulmonary tumor

	Cases	30-Day mortality				Hospital mortality		VATS
		Hospital		After discharge				
Primary malignant pulmonary tumor	45,243	107 (0.2)		28 (0.1)		244 (0.5)		34,249
Lung cancer	44,859	107 (0.2)		28 (0.1)		242 (0.5)		34,249
Adenocarcinoma	31,720	52 (0.2)		11 (0.03)		92 (0.3)		
Squamous cell carcinoma	8265	40 (0.5)		13 (0.2)		106 (1.3)		
Large cell carcinoma	280	0		0		1 (0.4)		
LCNEC	543	2 (0.4)		0		4 (0.7)		
Small cell carcinoma	785	3 (0.4)		2 (0.3)		10 (1.3)		
Adenosquamous carcinoma	560	2 (0.4)		0		4 (0.7)		
Carcinoma with pleomorphic, sarcomatoid or sarcomatous elements	511	4 (0.8)		0		10 (2.0)		
Carcinoid	252	0		0		0		
Carcinomas of salivary-gland type	40	0		0		1 (2.5)		
Unclassified	46	0		0		0		
Multiple lung cancer	1554	2 (0.1)		2 (0.1)		9 (0.6)		
Others	302	2 (0.7)		0		5 (1.7)		
Wedge resection	7683	11 (0.1)		13 (0.2)		21 (0.3)		6900
Segmental excision	5136	5 (0.1)		1 (0.02)		17 (0.3)		4219
*(Sleeve segmental excision)*	12	0		0		0		6
Lobectomy	31,365	83 (0.3)		14 (0.04)		188 (0.6)		22,880
*(Sleeve lobectomy)*	474	1 (0.2)		0		3 (0.6)		75
Pneumonectomy	324	5 (1.5)		0		10 (3.1)		42
*(Sleeve pneumonectomy)*	9	0		0		0		0
Other bronchoplasty	34	1 (2.9)		0		1 (2.9)		7
Pleuropneumonectomy	2	0		0		0		0
Others	315	2 (0.6)		0		5 (1.6)		201
Unknown	0	0		0		0		
Sarcoma	51	0		0		1 (2.0)		
AAH	103	0		0		0		
Others	230	0		0		1 (0.4)		

(), mortality %

**Table 12:** Details of lung cancer operations

TNM	
c-Stage	Cases
IA1	7,832
IA2	12,773
IA3	8,048
IB	4,977
IIA	1,577
IIB	3,862
IIIA	2,683
IIIB	499
IIIC	26
IVA	388
IVB	81
NA	2,113
Total	44,859

Sex	Cases
Male	27,385
Female	17,474
NA	0
Total	44,859

Cause of death	Cases
Cardiovascular	24
Pneumonia	41
Pyothorax	2
Bronchopleural fistula	13
Respiratory failure	22
Pulmonary embolism	5
Interstitial pneumonia	79
Brain infarction or bleeding	13
Others	65
Unknown	6
Total	270

p-Stage	Cases
0 (pCR)	3,234
IA1	9,035
IA2	9,839
IA3	4,890
IB	6,107
IIA	1,190
IIB	4,561
IIIA	3,808
IIIB	820
IIIC	16
IVA	1,010
IVB	73
NA	276
Total	44,859

Age (years)	Cases
< 20	25
20–29	31
30–39	277
40–49	1,195
50–59	3,736
60–69	13,290
70–79	20,190
80–89	6,003
≥ 90	112
NA	0
Total	44,859

**Table 13:** Metastatic pulmonary tumor

	Cases	30-Day mortality		Hospital mortality	VATS
		Hospital	After discharge		
Metastatic pulmonary tumor	8978	6 (0.1)	4 (0.04)	13 (0.1)	8342
Colorectal	4396	2 (0.05)	1 (0.02)	5 (0.1)	4088
Hepatobiliary/Pancreatic	433	0	0	0	414
Uterine	504	0	1 (0.2)	0	469
Mammary	543	2 (0.4)	0	3 (0.6)	522
Ovarian	82	0	0	0	76
Testicular	60	0	0	0	56
Renal	690	0	0	0	646
Skeletal	110	0	0	0	96
Soft tissue	261	0	0	0	238
Otorhinolaryngological	471	0	1 (0.2)	0	442
Pulmonary	470	1 (0.2)	0	2 (0.4)	405
Others	958	1 (0.1)	1 (0.1)	3 (0.3)	890

(), mortality %

**Table 14:** Tracheal tumor

	Cases	30-Day mortality		Hospital mortality
		Hospital	After discharge	
Tracheal tumor	127	5 (3.9)	1 (0.8)	6 (4.7)
A. Primary malignant tumor				
Histological classification				
Squamous cell carcinoma	17	1 (5.9)	0	1 (5.9)
Adenoid cystic carcinoma	17	0	0	0
Mucoepidermoid carcinoma	6	0	0	0
Others	19	0	0	1 (5.3)
Total	59	1 (1.7)	0	2 (3.4)
B. Metastatic/invasive malignant tumor, e.g. invasion of thyroid cancer	33	1 (3.0)	1 (3.0)	1 (3.0)
C. Benign tracheal tumor				
Histological classification				
Papilloma	1	0	0	0
Adenoma	2	0	0	0
Neurofibroma	1	0	0	0
Chondroma	0	0	0	0
Leiomyoma	3	0	0	0
Others	28	3 (10.7)	0	3 (10.7)
Histology unknown	0	0	0	0
Total	35	3 (8.6)	0	3 (8.6)
Operation				
Sleeve resection with reconstruction	30	0	0	0
Wedge with simple closure	1	0	0	0
Wedge with patch closure	1	0	0	0
Total laryngectomy with tracheostomy	0	0	0	0
Others	3	0	0	0
Unknown	0	0	0	0
Total	35	0	0	0

(), mortality %

**Table 15:** Tumor of pleural origin

Histological classification	Cases	30-Day mortality		Hospital mortality
		Hospital	After discharge	
Solitary fibrous tumor	146	0	0	0
Diffuse malignant pleural mesothelioma	264	4 (1.5)	1 (0.4)	13 (4.9)
Localized malignant pleural mesothelioma	32	0	0	1 (3.1)
Others	222	1 (0.5)	0	4 (1.8)
Total	664	5 (0.8)	1 (0.2)	18 (2.7)

Operative procedure	Cases	30-Day mortality		Hospital mortality
		Hospital	After discharge	
Extrapleural pneumonectomy	64	2 (3.1)	0	6 (9.4)
Total pleurectomy	100	1 (1.0)	0	2 (2.0)
Others	100	1 (1.0)	1 (1.0)	5 (5.0)
Total	264	4 (1.5)	1 (0.4)	13 (4.9)

(), mortality %

**Table 16:** Chest wall tumor

	Cases	30-Day mortality		Hospital mortality	VATS
		Hospital	After discharge		
Primary malignant tumor	132	0	0	0	71
Metastatic malignant tumor	179	1 (0.6)	0	1 (0.6)	84
Benign tumor	345	0	0	0	265
Total	656	1 (0.2)	0	1 (0.2)	420

(), mortality %

**Table 17:** Mediastinal tumor

	Cases	30-Day mortality		Hospital mortality	By VATS
		Hospital	After discharge		
Mediastinal tumor	5361	2 (0.04)	1 (0.02)	6 (0.1)	4009
Thymoma*	2098	0	0	2 (0.1)	1379
Thymic cancer	325	1 (0.3)	0	1 (0.3)	171
Thymus carcinoid	43	0	0	0	22
Germ cell tumor	81	0	0	0	44
*Benign*	58	0	0	0	35
*Malignant*	23	0	0	0	9
Neurogenic tumor	492	1 (0.2)	0	1 (0.2)	461
Congenital cyst	1224	0	0	0	1129
Goiter	98	0	0	1 (1.0)	40
Lymphatic tumor	172	0	0	1 (0.6)	122
Excision of pleural recurrence of thymoma	20	0	0	0	15
Thymolipoma	20	0	0	0	17
Others	788	0	1 (0.1)	0	609

(), mortality %

**Table 18:** Thymectomy for myasthenia gravis

	Cases	30-Day mortality		Hospital mortality	By VATS
		Hospital	After discharge		
Thymectomy for myasthenia gravis	499	0	0	0	319
With thymoma	348	0	0	0	209

(), mortality %

**Table 19:** Operations for non-neoplastic diseases

	Cases	30-Day mortality				Hospital mortality	
		Hospital		After discharge			
Operations for non-neoplastic diseases	22,996	229	(1.0)	30	(0.1)	465	(2.0)

	Cases	30-Day mortality			Hospital mortality	VATS
		Hospital		After discharge		
A. Inflammatory pulmonary disease	2400	8 (0.3)		4 (0.2)	18 (0.8)	2102
Tuberculous infection	54	1 (1.9)		0	1 (1.9)	46
Mycobacterial infection	526	2 (0.4)		0	3 (0.6)	465
Fungal infection	325	1 (0.3)		2 (0.6)	6 (1.8)	241
Bronchiectasis	64	0		0	0	48
Tuberculous nodule	70	0		0	0	65
Inflammatory pseudotumor	902	0		0	3 (0.3)	838
Interpulmonary lymph node	59	0		0	0	58
Others	400	4 (1.0)		2 (0.5)	5 (1.3)	341

(), mortality %

**Table 20:** B. Empyema

	Cases	30-day mortality		Hospital mortality	by VATS
		hospital	After discharge		
Acute empyema	2402	57 (2.4)	4 (0.2)	124 (5.2)	2013
With fistula	509	34 (6.7)	1 (0.2)	66 (13.0)	270
Without fistula	1876	22 (1.2)	3 (0.2)	54 (2.9)	1729
Unknown	17	1 (5.9)	0	4 (23.5)	14
Chronic empyema	701	23 (3.3)	1 (0.1)	63 (9.0)	407
With fistula	325	14 (4.3)	0	36 (11.1)	125
Without fistula	324	8 (2.5)	1 (0.3)	25 (7.7)	241
Unknown	52	1 (1.9)	0	2 (3.8)	41
Total	3103	80 (2.6)	5 (0.2)	187 (6.0)	2420

(), mortality %

**Table 21:** C. Descending necrotizing mediastinitis

	Cases	30-day mortality		Hospital mortality	VATS
		Hospital	After discharge		
C. Descending necrotizing mediastinitis	106	4 (3.8)	1 (0.9)	6 (5.7)	81

(), mortality %

**Table 22:** D. Bullous diseases

	Cases	30-Day mortality		Hospital mortality	VATS
		Hospital	After discharge		
D. Bullous diseases	376	0	0	0	349
Emphysematous bulla	270	0	0	0	258
Bronchogenic cyst	21	0	0	0	18
Emphysema with LVRS	23	0	0	0	19
Others	62	0	0	0	54

(), mortality %

*LVRS* lung volume reduction surgery

**Table 23:** E. Pneumothorax

Cases	30-day mortality		Hospital mortality	VATS
	Hospital	After discharge		
14,731	81 (0.5)	19 (0.1)	146 (1.0)	14,379
Spontaneous pneumothorax				

Operative procedure	Cases	30-Day mortality		Hospital mortality	VATS
		Hospital	After discharge		
Bullectomy	2825	7 (0.2)	4 (0.1)	11 (0.4)	2,770
Bullectomy with additional procedure	7632	5 (0.1)	1 (0.01)	10 (0.1)	7,535
Coverage with artificial material	7383	5 (0.1)	1 (0.01)	10 (0.1)	7,291
Parietal pleurectomy	27	0	0	0	27
Coverage and parietal pleurectomy	57	0	0	0	54
Others	165	0	0	0	163
Others	657	3 (0.5)	2 (0.3)	5 (0.8)	610
Unknown	10	0	0	0	9
Total	11,124	15 (0.1)	7 (0.1)	26 (0.2)	10,924
Secondary pneumothorax					

Associated disease	Cases	30-Day mortality		Hospital mortality	VATS
		Hospital	After discharge		
COPD	2,437	33 (1.4)	8 (0.3)	65 (2.7)	2,359
Tumorous disease	128	9 (7.0)	1 (0.8)	14 (10.9)	123
Catamenial	199	0	0	1 (0.5)	194
LAM	39	0	0	0	38
Others (excluding pneumothorax by trauma)	804	24 (3.0)	3 (0.4)	40 (5.0)	741
Unknown	0	0	0	0	0

Operative procedure	Cases	30 Day mortality		Hospital mortality	VATS
		Hospital	After discharge		
Bullectomy	607	5 (0.8)	1 (0.2)	8 (1.3)	587
Bullectomy with additional procedure	2,079	33 (1.6)	5 (0.2)	50 (2.4)	2,030
Coverage with artificial material	1,969	32 (1.6)	5 (0.3)	49 (2.5)	1,924
Parietal pleurectomy	4	0	0	0	4
Coverage and parietal pleurectomy	37	0	0	0	35
Others	69	1 (1.4)	0	1 (1.4)	67
Others	917	28 (3.1)	6 (0.7)	62 (6.8)	836
Unknown	4	0	0	0	2
Total	3607	66 (1.8)	12 (0.3)	120 (3.3)	3,455

(), mortality %

**Table 24:** F. Chest wall deformity

	Cases	30-Day mortality		Hospital mortality
		Hospital	After discharge	
F. Chest wall deformity	176	0	0	1 (0.6)
Funnel chest	165	0	0	1 (0.6)
Others	11	0	0	0

(), mortality %

**Table 25:** G. Diaphragmatic hernia

	Cases	30-Day mortality		Hospital mortality	VATS
		Hospital	After discharge		
G. Diaphragmatic hernia	30	1 (3.3)	0	3 (10.0)	21
Congenital	5	0	0	2 (40.0)	4
Traumatic	4	0	0	0	3
Others	21	1 (4.8)	0	1(4.8)	14

(), mortality %

**Table 26:** H. Chest trauma

	Cases	30-Day mortality		Hospital mortality	VATS
		Hospital	After discharge		
H. Chest trauma	431	21 (4.9)	0	33 (7.7)	280

(), mortality %

**Table 27:** I. Other respiratory surgery

	Cases	30-Day mortality		Hospital mortality	VATS
		Hospital	After discharge		
I. Other respiratory surgery	1643	34 (2.1)	1 (0.1)	71 (4.3)	1258
Arteriovenous malformation*	84	0	0	0	79
Pulmonary sequestration	103	0	0	0	92
Postoperative bleeding · air leakage	481	17 (3.5)	0	36 (7.5)	329
Chylothorax	73	0	0	3 (4.1)	61
Others	902	17 (1.9)	1 (0.1)	32 (3.5)	697

(), mortality %

In 2018, a total of 2342 procedures for benign pulmonary tumors had been conducted (Table 10). Hamartomas were the most frequent benign pulmonary tumors diagnosed, with 2222 patients (95%) undergoing VATS.

Additional information on primary malignant pulmonary tumors is shown in Tables 11, 12. Accordingly, adenocarcinoma had been the most frequently diagnosed lung cancer subtype (71% of all lung cancers), followed by squamous cell carcinoma (18%). Sublobar resection was performed in 12,819 lung cancer cases (29% of all cases) and lobectomy in 31,365 cases (70% of all cases). Sleeve lobectomy was performed in 474 cases, while pneumonectomy was required in 324 cases (0.7% of all cases). VATS lobectomy for lung cancer was performed in 22,880 cases (73% of all lobectomy cases). The number of patients aged 80 years or older who underwent lung cancer surgery was 6115 (14%). Among those who died within 30 days following surgery, 107 died prior to hospital discharge, while 28 died after discharge. Overall, 135 patients died within 30 days after surgery (30-day mortality rate, 0.3%), while 242 died prior to discharge (hospital mortality rate, 0.5%). Moreover, 30-day mortality rates according to procedure were 0.1%, 0.2%, and 1.5% for segmentectomy, lobectomy, and pneumonectomy, respectively. Interstitial pneumonia had been the leading cause of death following lung cancer surgery, followed by pneumonia, cardiovascular events, and respiratory failure.

The procedures for metastatic pulmonary tumors, 8978 of which were performed in 2018, are shown in Table 13. Among such procedures, colorectal cancer had been the most frequent diagnosis (49% of all cases).

A total of 59 procedures for malignant tracheal tumor were performed in 2018; however, 30 patients underwent sleeve resection and reconstruction (Table 14).

Overall, 664 pleural tumors had been diagnosed in 2018 (Table 15), with diffuse malignant pleural mesothelioma being the most frequent histologic diagnosis. Total pleurectomy was performed in 100 cases and extrapleural pneumonectomy in 64 cases. The 30-day mortality rate was 1% and 3% following total pleurectomy and extrapleural pneumonectomy, respectively, both of which had better outcomes than previously reported.

Overall, 656 chest wall tumor resections had been performed in 2018 (Table 16), among which 345 (53%) were benign. Among the 311 malignant chest wall tumors, 179 (58%) were metastatic.

A total of 5361 mediastinal tumors were resected in 2018, a slight increase compared to that in the previous year (Table 17). Thymic epithelial tumors—including 2098 thymomas, 325 thymic carcinomas, and 43 thymic carcinoids—were the most frequently diagnosed mediastinal tumor subtype in 2018.

In total, 499 patients underwent thymectomy for myasthenia gravis (Table 18), among which 348 procedures were associated with thymoma.

Overall, 22,996 patients underwent procedures for non-neoplastic disease. Accordingly, 2400 patients underwent lung resection for inflammatory lung diseases (Table 19), among which 22% and 14% were associated with mycobacterial infections and fungal infections, respectively. Procedures for inflammatory nodules were performed in cases where lung cancer was suspected prior to surgery (902 cases, 38%).

A total of 3103 procedures were performed for empyema (Table 20), among which 2402 (77%) were acute and 701 were chronic. Moreover, 509 patients with acute empyema and 325 patients with chronic empyema had developed bronchopleural fistulas. The hospital mortality rate was 13% among patients with acute empyema with fistula.

In 2018, 106 operations were performed for descending necrotizing mediastinitis (Table 21), with a hospital mortality rate of 6%. Furthermore, 376 procedures were conducted for bullous diseases (Table 22), while only 23 patients underwent lung volume reduction surgery.

A total of 14,731 procedures were performed for spontaneous pneumothorax (Table 23). Among the 11,124 procedures for primary pneumothorax, 2825 (25%) were bullectomies alone, while 7632 (69%) required additional procedures. A total of 3607 procedures for secondary pneumothorax were conducted, with COPD being the most prevalent associated disease (2437 cases, 68%). The hospital mortality rate for secondary pneumothorax associated with COPD was 2.7%.

The 2018 survey reported 176 procedures for chest wall deformity (Table 24). However, this may have been underestimated given that the Nuss procedure for pectus excavatum was more likely to have been performed in pediatric surgery centers not associated with the Japanese Association for Thoracic Surgery.

Overall, 30 patients underwent surgical treatment for diaphragmatic hernia (Table 25). This figure may have also been underestimated considering that procedures may have been classified as gastrointestinal surgery.

The survey reported 431 procedures for chest trauma, excluding iatrogenic injuries (Table 26), with a hospital mortality rate of 8%.

Table 27 summarizes the procedures for other diseases, including 84 and 103 cases of arteriovenous malformation and pulmonary sequestration, respectively.

A total of 71 lung transplantations were performed in 2018 (Table 28), among which 57 and 14 were from brain-dead and living related donors, respectively.

**Table 28:** Lung transplantation

	Cases	30-Day mortality		Hospital mortality
		Hospital	After discharge	
Single lung transplantation from brain-dead donor	30	0	0	0
Bilateral lung transplantation from brain-dead donor	27	0	0	0
Lung transplantation from living donor	14	0	0	1 (7.1)
Total lung transplantation	71	0	0	1 (1.4)
Donor of living donor lung transplantation	23	0	0	0

(), mortality %

The number of VATS procedures has continued to increase annually, ultimately reaching 71,171 (82% of all general thoracic surgeries) in 2018 (Table 29).

**Table 29:** Video-assisted thoracic surgery

	Cases	30-Day mortality		Hospital mortality
		hospital	After discharge	
Video-assisted thoracic surgery	71,171	229 (0.3)	48 (0.1)	474 (0.7)

(), mortality % (including thoracic sympathectomy 160)

Details regarding tracheobronchoplasty, pediatric surgery, and combined resection of neighboring organs are presented in Tables 30, 31, 32, 33.

**Table 30:** Tracheobronchoplasty

	Cases	30-Day mortality		Hospital mortality
		Hospital	After discharge	
Tracheobronchoplasty	747	8 (1.1)	1 (0.1)	11 (1.5)
Trachea	46	1 (2.2)	0	1 (2.2)
Sleeve resection with reconstruction	32	0	0	0
Wedge with simple closure	4	0	0	0
Wedge with patch closure	1	0	0	0
Total laryngectomy with tracheostomy	0	0	0	0
Others	9	1 (11.1)	0	1 (11.1)
Carinal reconstruction	35	0	0	1 (2.9)
Sleeve pneumonectomy	10	0	0	0
Sleeve lobectomy	464	1 (0.2)	0	2 (0.4)
Sleeve segmental excision	15	0	0	0
Bronchoplasty without lung resection	23	1 (4.3)	1 (4.3)	1 (4.3)
Others	154	5 (3.2)	0	6 (3.9)

(), mortality %

**Table 31:** Pediatric surgery

	Cases	30-Day mortality		Hospital mortality
		Hospital	After discharge	
Pediatric surgery	287	7 (2.4)	1 (0.3)	11 (3.8)

(), mortality %

**Table 32:** Combined resection of neighboring organ(s)

	Cases	30-Day mortality				Hospital mortality	
		Hospital		After discharge			
Combined resection of neighboring organ(s)	1401	10	(0.7)	1	(0.1)	21	(1.5)

Organ resected	Cases	30-Day mortality		Hospital mortality
		Hospital	After discharge	
A. Primary lung cancer				
Aorta	10	1 (10.0)	0	1 (10.0)
Superior vena cava	21	0	0	1 (4.8)
Brachiocephalic vein	8	0	0	1 (12.5)
Pericardium	122	1 (0.8)	0	4 (3.3)
Pulmonary artery	146	1 (0.7)	0	2 (1.4)
Left atrium	18	0	0	0
Diaphragm	74	1 (1.4)	0	1 (1.4)
Chest wall (including ribs)	330	5 (1.5)	0	9 (2.7)
Vertebra	8	0	0	0
Esophagus	4	0	0	0
Total	741	9 (1.2)	0	19 (2.6)
B. Mediastinal tumor				
Aorta	6	1 (16.7)	0	1 (16.7)
Superior vena cava	53	0	0	1 (1.9)
Brachiocephalic vein	112	0	0	1 (0.9)
Pericardium	336	0	1 (0.3)	1 (0.3)
Pulmonary artery	4	1 (25.0)	0	1 (25.0)
Left atrium	2	0	0	0
Diaphragm	30	0	0	0
Chest wall (including ribs)	4	0	0	0
Vertebra	5	0	0	0
Esophagus	4	0	0	0
Lung	487	1 (0.2)	1 (0.2)	2 (0.4)
Total	1,043	3 (0.3)	2 (0.2)	7 (0.7)

(), mortality %

**Table 33:** Operation of lung cancer invading the chest wall of the apex

	Cases	30-Day mortality		Hospital mortality
		Hospital	After discharge	
15. Operation of lung cancer invading the chest wall of the apex	772	6 (0.8)	0	9 (1.2)

(), mortality %

Includes tumors invading the anterior apical chest wall and posterior apical chest wall (superior sulcus tumor, so called Pancoast type)

### (C) Esophageal surgery

In 2018, the data collection method for esophageal surgery had been modified from self-reports using questionnaire sheets according to each institution belonging to the Japanese Association for Thoracic Surgery to an automatic package downloaded from the NCD in Japan. Consequently, data for non-surgical cases with esophageal diseases had been excluded from the registry. Furthermore, data regarding the histological classification of malignant tumors, multiple primary cancers, and mortality rates for cases with combined resection of other organs could not be registered given that they were not included in the NCD. Instead, detailed data regarding postoperative surgical and non-surgical complications were collected from the NCD. Moreover, data regarding surgeries for corrosive esophageal strictures and salvage surgeries for esophageal cancer had been exceptionally registered by participating institutions.

**Fig. 3:**
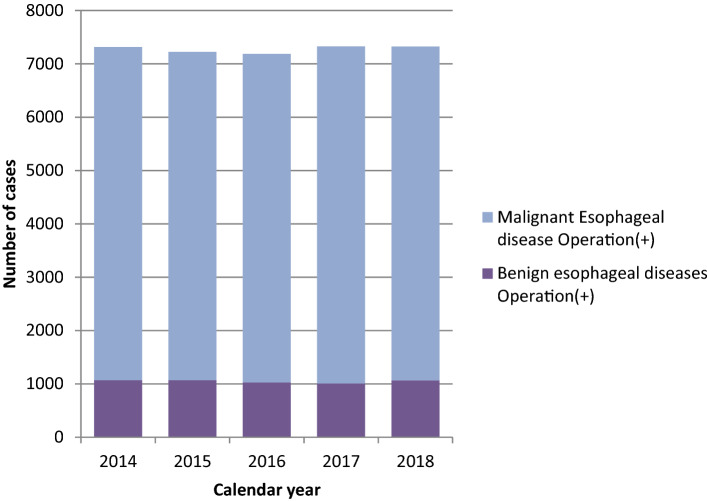
Annual trend of in-patients with esophageal diseases

Throughout 2018, a total of 7324 patients underwent surgery for esophageal diseases (1068 and 6256 for benign and malignant esophageal diseases, respectively) from 552 institutions across Japan. Among them, 329 (63.0%) and 441 (79.9%) institutions performed surgeries for benign and malignant esophageal diseases, respectively. Among institutions performing surgeries for malignant esophageal diseases, 82 (18.6%) had 20 or more patients who underwent esophageal surgeries within 2018, while 271 (61.5%) had less than 10 patients (i.e., 1–9 patients) who underwent the same procedure within the same year. This distribution was quite different from that in 2017 [125 (29.2%) and 215 (50.2%), respectively], suggesting the differences between the two data collection methods, as mentioned previously (Table 34). Annual trends among registered in-patients with esophageal diseases have remained unchanged for the past 5 years (Fig. 3).

**Table 34:** Distribution of number of esophageal operations in 2018 in each institution

Esophageal surgery			
Number of operations in 2018	Benign esophageal diseases	Malignant Esophageal disease	Benign + Malignant
0	224	111	63
1–4	271	179	193
5–9	43	92	101
10–19	10	88	95
20–29	0	36	38
30–39	1	10	21
40–49	2	13	14
≧ 50	1	23	27
Total	552	552	552

With regard to benign esophageal diseases (Table 35), thoracoscopic and/or laparoscopic surgeries were performed in 89.1% (115/129), 81.5% (387/475), 51.6% (33/64), and 49.5% (102/206) of patients with esophagitis (including esophageal ulcer), hiatal hernia, benign tumors, and achalasia, respectively. On the other hand, 89.1% (115/129) of patients with spontaneous rupture of the esophagus underwent open surgery. Hospital mortality rates after surgery for benign esophageal diseases had only been recorded for those with hiatal hernia and spontaneous rupture of the esophagus, with 8 (1.7%) and 2 (1.6%) patients succumbing to mortality within 30 days following surgery, respectively. Only 3 (0.4%) among the 673 patients who underwent thoracoscopic and/or laparoscopic surgery died within 30 postoperative days, all of whom had hiatal hernia.

**Table 35:** Benign esophageal diseases

	Operation ( +)				T/L*3			
	Cases	Hospital mortality			Cases	Hospital mortality		
		~ 30 days	31–90 days	Total (including after 91 days mortality)		~ 30 days	31–90 days	Total (including after 91 days mortality)
1. Achalasia	206	0	0	0	102	0	0	0
2. Benign tumor	64	0	0	0	33	0	0	0
3. Diverticulum	41	0	0	0	13	0	0	0
4. Hiatal hernia	475	8 (1.7)	5 (1.1)	13 (2.7)	387	3 (0.8)	3 (0.8)	6 (1.6)
5. Spontaneous rupture of the esophagus	129	2 (1.6)	0	2 (1.6)	14	0	0	0
6. Esophago-tracheal fistula	2	0	0	0	1	0	0	0
7. Esophagitis, Esophageal ulcer	129	0	0	0	115	0	0	0
8. Corrosive stricture of the esophagus	22	0	0	0	8	0	0	0
Total	1068	10 (0.9)	5 (0.5)	15 (1.4)	673	3 (0.4)	3 (0.4)	6 (0.9)

(), mortality %

*T/L* Thoracoscopic and/or laparoscopic

The most common tumor location for malignant esophageal diseases was the thoracic esophagus (Table 36). Among 6256 cases with esophageal malignancies, 2538 (40.6%) and 3718 (59.4%) underwent esophagectomy for superficial and advanced cancers, respectively. The 30-day and hospital mortality rates following esophagectomy were 0.4% and 0.6% for patients with superficial cancer and 1.0% and 1.8% for those with advanced cancer, respectively.

**Table 36:** Malignant esophageal disease

	Operation (+)					Thoracoscopic and/or laparscopic procedure				
	Cases	Hospital mortality				Cases	Conversion to thoracotomy	Hospital mortality		
		~ 30 days		31–90 days	Total (including after 91days mortality)			~ 30 days	31–90 days	Total (including after 91days mortality)
Location										
(1) Cervical esophagus	172	2 (1.2)		2 (1.2)	3 (1.7)	67	0	1 (1.5)	1 (1.5)	2 (3.0)
(2) Thoracic esophagus	5244	41 (0.8)		27 (0.5)	69 (1.3)	3848	42 (1.1)	28 (0.7)	15 (0.4)	43 (1.1)
(3) Abdominal esophagus	499	1 (0.2)		2 (0.4)	3 (0.6)	272	2 (0.7)	1 (0.4)	0	1 (0.4)
Total	5915	44 (0.7)		31 (0.5)	75 (1.3)	4187	44 (1.1)	30 (0.7)	16 (0.4)	46 (1.1)
Tumor depth										
(A) Superficial cancer (T1)										
(1) Transhiatal esophagectomy	15	0		0	0	0	0	0	0	0
(2) Mediastinoscopic esophagectomy and reconstruction	90	0		0	0	90	0	0	0	0
(3) Transthoracic (rt.) esophagectomy and reconstruction	1908	8 (0.4)		6 (0.3)	14 (0.7)	1534	12 (0.8)	7 (0.5)	4 (0.3)	11 (0.7)
(4) Transthoracic (lt.) esophagectomy and reconstruction	43	0		1 (2.3)	1 (2.3)	19	0	0	0	0
(5) Cervical esophageal resection and reconstruction	19	0		0	0	0	0	0	0	0
(6) Robot-assisted esophagectomy and reconstruction	172	1 (0.6)		0	1 (0.6)	174	0	2 (1.1)	0	2 (1.1)
(7) Others	58	0		0	0	14	1 (7.1)	0	0	0
(8) Esophagectomy without reconstruction	233	0		0	0	1	0	0	0	0
Subtotal	2538	9 (0.4)		7 (0.3)	16 (0.6)	1832	13 (0.7)	9 (0.5)	4 (0.2)	13 (0.7)
(B) Advanced cancer (T2–T4)										
(1) Transhiatal esophagectomy	32	1 (3.1)		0	1 (3.1)	0	0	0	0	0
(2) Mediastinoscopic esophagectomy and reconstruction	83	2 (2.4)		0	2 (2.4)	82	0	2 (2.4)	0	2 (2.4)
(3) Transthoracic (rt.) esophagectomy and reconstruction	3045	30 (1.0)		18 (0.6)	49 (1.6)	2017	29 (1.4)	19 (0.9)	10 (0.5)	29 (1.4)
(4) Transthoracic (lt.) esophagectomy and reconstruction	94	0		1 (1.1)	1 (1.1)	25	0	0	0	0
(5) Cervical esophageal resection and reconstruction	66	0		1 (1.5)	0	0	0	0	0	0
(6) Robot-assisted esophagectomy and reconstruction	156	0		0	0	156	1 (0.6)	0	0	0
(7) Others	92	1 (1.1)		1 (1.1)	2 (2.2)	23	0	0	0	0
(8) Esophagectomy without reconstruction	150	4 (2.7)		8 (5.3)	12 (8.0)	8	0	0	2 (25.0)	2 (25.0)
Subtotal	3718	38 (1.0)		29 (0.8)	67 (1.8)	2311	30 (1.3)	21 (0.9)	12 (0.5)	33 (1.4)
Total	6256	47 (0.8)		36 (0.6)	83 (1.3)	4143	43 (1.0)	30 (0.7)	16 (0.4)	46 (1.1)

	Cases	Overall morbidity		Morbidity ≥ CD III	Surgical complications					
					Surgical site infection			Anastomotic leakage	Recurrent nerve palsy	Wound dehiscence
					Superficial incision	Deep incision	Organ space			
Location										
(1) Cervical esophagus	172	105 (61.0)		49 (28.5)	13 (7.6)	5 (2.9)	11 (6.4)	17 (9.9)	14 (8.1)	3 (1.7)
(2) Thoracic esophagus	5244	3061 (58.4)		1188 (22.7)	360 (6.9)	206 (3.9)	463 (8.8)	738 (14.1)	785 (15.0)	66 (1.3)
(3) Abdominal esophagus	499	236 (47.3)		93 (18.6)	21 (4.2)	11 (2.2)	43 (8.6)	65 (13.0)	29 (5.8)	4 (0.8)
Total	5915	3402 (57.5)		1330 (22.5)	394 (6.7)	222 (3.8)	517 (8.7)	820 (13.9)	828 (14.0)	73 (1.2)
Tumor depth										
(A) Superficial cancer (T1)										
(1) Transhiatal esophagectomy	15	10	(66.7)	7 (46.7)	2 (13.3)	2 (13.3)	3 (20.0)	5 (33.3)	1 (6.7)	0
(2) Mediastinoscopic esophagectomy and reconstruction	90	54	(60.0)	20 (22.2)	7 (7.8)	2 (2.2)	8 (8.9)	18 (20.0)	22 (24.4)	0
(3) Transthoracic (rt.) esophagectomy and reconstruction	1908	1100	(57.7)	421 (22.1)	129 (6.8)	75 (3.9)	171 (9.0)	294 (15.4)	274 (14.4)	29 (1.5)
(4) Transthoracic (lt.) esophagectomy and reconstruction	43	19	(44.2)	9 (20.9)	2 (4.7)	1 (2.3)	4 (9.3)	5 (11.6)	1 (2.3)	0
(5) Cervical esophageal resection and reconstruction	19	15	(78.9)	3 (15.8)	1 (5.3)	0	1 (5.3)	2 (10.5)	3 (15.8)	0
(6) Robot-assisted esophagectomy and reconstruction	172	99 (57.6)		35 (20.3)	6 (3.5)	2 (1.2)	13 (7.6)	25 (14.5)	25 (14.5)	1 (0.6)
(7) Others	58	30 (51.7)		9 (15.5)	0	0	5 (8.6)	11 (19.0)	0	0
(8) Esophagectomy without reconstruction	233	32 (13.7)		10 (4.3)	0	0	0	0	0	0
Subtotal	2538	1359 (53.5)		514 (20.3)	147 (5.8)	82 (3.2)	205 (8.1)	360 (14.2)	326 (12.8)	30 (1.2)
(B) Advanced cancer (T2–T4)										
(1) Transhiatal esophagectomy	32	18 (56.3)		9 (28.1)	0	4 (12.5)	4 (12.5)	6 (18.8)	2 (6.3)	0
(2) Mediastinoscopic esophagectomy and reconstruction	83	55 (66.3)		21 (25.3)	4 (4.8)	2 (2.4)	6 (7.2)	16 (19.3)	20 (24.1)	0
(3) Transthoracic (rt.) esophagectomy and reconstruction	3045	1749 (57.4)		696 (22.9)	221 (7.3)	121 (4.0)	266 (8.7)	386 (12.7)	422 (13.9)	38 (1.2)
(4) Transthoracic (lt.) esophagectomy and reconstruction	94	51 (54.3)		23 (24.5)	4 (4.3)	6 (6.4)	9 (9.6)	10 (10.6)	7 (7.4)	0
(5) Cervical esophageal resection and reconstruction	66	41 (62.1)		15 (22.7)	9 (13.6)	3 (4.5)	1 (1.5)	4 (6.1)	7 (10.6)	0
(6) Robot-assisted esophagectomy and reconstruction	156	87 (55.8)		25 (16.0)	6 (3.8)	2 (1.3)	7 (4.5)	17 (10.9)	36 (23.1)	0
(7) Others	92	45 (48.9)		19 (20.7)	0	0	11 (12.0)	13 (14.1)	4 (4.3)	2 (2.2)
(8) Esophagectomy without reconstruction	150	77 (51.3)		38 (25.3)	1 (0.7)	0	2 (1.3)	0	0	1 (0.7)
Subtotal	3718	2123 (57.1)		846 (22.8)	245 (6.6)	138 (3.7)	306 (8.2)	452 (12.2)	498 (13.4)	41 (1.1)
Total	6256	3482 (55.7)		1360 (21.7)	392 (6.3)	220 (3.5)	511 (8.2)	812 (13.0)	824 (13.2)	71 (1.1)

	Cases	Nonsurgical complications									Readmission within 30 days	Reoperation within 30 days
		Pneumonia	Unplanned intubation	prolonged ventilation>48h	pulmonary embolism	atelectasis	Renal failure	CNS events	Cardiac events	Septic shock		
Location												
(1) Cervical esophagus	172	16 (9.3)	12 (7.0)	18 (10.5)	5 (2.9)	7 (4.1)	3 (1.7)	1 (0.6)	3 (1.7)	3 (1.7)	3 (1.7)	17 (9.9)
(2) Thoracic esophagus	5244	798 (15.2)	269 (5.1)	337 (6.4)	38 (0.7)	302 (5.8)	27 (0.5)	27 (0.5)	20 (0.4)	38 (0.7)	134 (2.6)	333 (6.4)
(3) Abdominal esophagus	499	56 (11.2)	17 (3.4)	22 (4.4)	2 (0.4)	33 (6.6)	3 (0.6)	2 (0.4)	1 (0.2)	6 (1.2)	16 (3.2)	29 (5.8)
Total	5915	870 (14.7)	298 (5.0)	377 (6.4)	45 (0.8)	342 (5.8)	33 (0.6)	30 (0.5)	24 (0.4)	47 (0.8)	153 (2.6)	379 (6.4)
Tumor depth												
(A) Superficial cancer (T1)												
(1) Transhiatal esophagectomy	15	5 (33.3)	0	0	0	2 (13.3)	0	0	0	1 (6.7)	1 (6.7)	3 (20.0)
(2) Mediastinoscopic esophagectomy and reconstruction	90	10 (11.1)	6 (6.7)	8 (8.9)	0	3 (3.3)	1 (1.1)	0	1 (1.1)	1 (1.1)	2 (2.2)	5 (5.6)
(3) Transthoracic (rt.) esophagectomy and reconstruction	1908	281 (14.7)	84 (4.4)	105 (5.5)	20 (1.0)	97 (5.1)	10 (0.5)	9 (0.5)	6 (0.3)	13 (0.7)	44 (2.3)	123 (6.4)
(4) Transthoracic (lt.) esophagectomy and reconstruction	43	4 (9.3)	2 (4.7)	3 (7.0)	0	3 (7.0)	0	0	0	0	1 (2.3)	2 (4.7)
(5) Cervical esophageal resection and reconstruction	19	3 (15.8)	1 (5.3)	1 (5.3)	0	2 (10.5)	0	1 (5.3)	0	0	0	1 (5.3)
(6) Robot-assisted esophagectomy and reconstruction	172	21 (12.2)	5 (2.9)	9 (5.2)	3 (1.7)	3 (1.7)	3 (1.7)	2 (1.2)	0	0	3 (1.7)	3 (1.7)
(7) Others	58	7 (12.1)	1 (1.7)	1 (1.7)	1 (1.7)	5 (8.6)	0	0	0	0	2 (3.4)	4 (6.9)
(8) Esophagectomy without reconstruction	233	0	0	0	0	0	0	0	0	0	4 (1.7)	0
Subtotal	2538	331 (13.0)	99 (3.9)	127 (5.0)	24 (0.9)	115 (4.5)	14 (0.6)	12 (0.5)	7 (0.3)	15 (0.6)	57 (2.2)	141 (5.6)
(B) Advanced cancer (T2–T4)												
(1) Transhiatal esophagectomy	32	1 (3.1)	1 (3.1)	3 (9.4)	0	1 (3.1)	0	0	1 (3.1)	0	0	3 (9.4)
(2) Mediastinoscopic esophagectomy and reconstruction	83	7 (8.4)	3 (3.6)	6 (7.2)	1 (1.2)	2 (2.4)	1 (1.2)	0	1 (1.2)	1 (1.2)	1 (1.2)	5 (6.0)
(3) Transthoracic (rt.) esophagectomy and reconstruction	3045	474 (15.6)	177 (5.8)	213 (7.0)	15 (0.5)	200 (6.6)	15 (0.5)	14 (0.5)	13 (0.4)	26 (0.9)	89 (2.9)	202 (6.6)
(4) Transthoracic (lt.) esophagectomy and reconstruction	94	15 (16.0)	4 (4.3)	6 (6.4)	2 (2.1)	6 (6.4)	1 (1.1)	2 (2.1)	0	0	5 (5.3)	6 (6.4)
(5) Cervical esophageal resection and reconstruction	66	6 (9.1)	4 (6.1)	3 (4.5)	1 (1.5)	0	0	1 (1.5)	1 (1.5)	0	0	5 (7.6)
(6) Robot-assisted esophagectomy and reconstruction	156	19 (12.2)	5 (3.2)	7 (4.5)	2 (1.3)	7 (4.5)	0	0	1 (0.6)	0	3 (1.9)	6 (3.8)
(7) Others	92	8 (8.7)	3 (3.3)	6 (6.5)	0	9 (9.8)	0	0	0	1 (1.1)	3 (3.3)	6 (6.5)
(8) Esophagectomy without reconstruction	150	3 (2.0)	2 (1.3)	3 (2.0)	0	1 (0.7)	0	1 (0.7)	0	2 (1.3)	6 (4.0)	3 (2.0)
Subtotal	3718	533 (14.3)	199 (5.4)	247 (6.6)	21 (0.6)	226 (6.1)	17 (0.5)	18 (0.5)	17 (0.5)	30 (0.8)	107 (2.9)	236 (6.3)
Total	6256	864 (13.8)	298 (4.8)	374 (6.0)	45 (0.7)	341 (5.5)	31 (0.5)	30 (0.5)	24 (0.4)	45 (0.7)	164 (2.6)	377 (6.0)

**Fig. 4:**
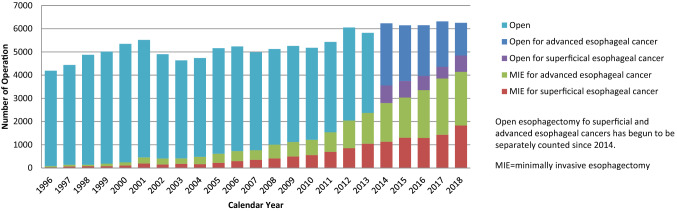
Annual trend of esophagectomy

Among esophagectomy procedures, transthoracic esophagectomy via right thoracotomy or right thoracoscopy was most commonly adopted for patients with a superficial cancer (1908/2538, 75.2%) and advanced cancer (3045/3718, 81.9%) (Table 36). Transhiatal esophagectomy, which is commonly performed in Western countries, was adopted in only 15 (0.6%) and 32 (0.9%) patients with superficial and advanced cancer who underwent esophagectomy in Japan, respectively. Thoracoscopic and/or laparoscopic esophagectomy was utilized in 1832 (72.2%) and 2311 (62.2%) patients with superficial and advanced cancer, respectively. The number of patients who underwent thoracoscopic and/or laparoscopic surgery for superficial or advanced cancer has been increasing, whereas that of open surgery, especially for advanced cancer, has been decreasing annually (Fig. 4). Mediastinoscopic and robot-assisted esophagectomy and reconstruction were performed for 173 and 328 patients in 2018, respectively. The 30-day and hospital mortality rates following thoracoscopic and/or laparoscopic esophagectomy were 0.5% and 0.7% for patients with superficial cancer and 0.9% and 1.4% or those with advanced cancer, respectively (Table 36).

Detailed data collection regarding postoperative surgical and non-surgical complications have been initiated this year (Table 36). Overall, 1360 (21.7%) of 6256 patients developed grade III or higher complications based on the Clavien–Dindo classification. Among surgical complications, anastomotic leakage and recurrent nerve palsy occurred in 13.0% and 13.2% of the patients and in approximately 20% and 24% of those who underwent mediastinoscopic esophagectomy, respectively. Among non-surgical postoperative complications, pneumonia occurred in 13.8% of the patients, 4.8% of whom underwent unplanned intubation. Mediastinoscopic esophagectomy seemed to be less likely to promote postoperative pneumonia compared to transthoracic (rt.) esophagectomy. Postoperative pulmonary embolism occurred in 0.7% of the patients.

Salvage surgery following definitive (chemo) radiotherapy was performed in 570 patients, with a 30-day and hospital mortality rate of 0.5% and 1.6%, respectively. Thoracoscopic and/or laparoscopic esophagectomy were performed in 272 (47.7%) patients, both of which had comparable mortality rates (Table 37).

**Table 37:** Salvage surgery

	Operation (+)				Thoracoscopic and/or laparscopic procedure					EMR or ESD
	Cases	Hospital mortality			Cases	Conversion to thoracotomy	Hospital mortality			
		~ 30 days	31–90 days	Total (including after 91days mortality)			~ 30 days	31–90days	Total (including after 91days mortality)	
Salvage surgery	570	3 (0.5)	6 (1.1)	9 (1.6)	272	4 (1.5)	1 (0.4)	3 (1.1)	4 (1.5)	245

We aim to continue our efforts in collecting comprehensive survey data through more active collaboration with the Japan Esophageal Society and other related institutions.
